# Structural basis for Z-DNA binding and stabilization by the zebrafish Z-DNA dependent protein kinase PKZ

**DOI:** 10.1093/nar/gkt743

**Published:** 2013-08-23

**Authors:** Matteo de Rosa, Sonia Zacarias, Alekos Athanasiadis

**Affiliations:** Instituto Gulbenkian de Ciência, Rua Quinta Grande 6, 2780-156 Oeiras, Portugal

## Abstract

The RNA-dependent protein kinase PKR plays a central role in the antiviral defense of vertebrates by shutting down protein translation upon detection of viral dsRNA in the cytoplasm. In some teleost fish, PKZ, a homolog of PKR, performs the same function, but surprisingly, instead of dsRNA binding domains, it harbors two Z-DNA/Z-RNA-binding domains belonging to the Zalpha domain family. Zalpha domains have also been found in other proteins, which have key roles in the regulation of interferon responses such as ADAR1 and DNA-dependent activator of IFN-regulatory factors (DAI) and in viral proteins involved in immune response evasion such as the poxviral E3L and the Cyprinid Herpesvirus 3 ORF112. The underlying mechanism of nucleic acids binding and stabilization by Zalpha domains is still unclear. Here, we present two crystal structures of the zebrafish PKZ Zalpha domain (DrZalpha^PKZ^) in alternatively organized complexes with a (CG)_6_ DNA oligonucleotide at 2 and 1.8 Å resolution. These structures reveal novel aspects of the Zalpha interaction with DNA, and they give insights on the arrangement of multiple Zalpha domains on DNA helices longer than the minimal binding site.

## INTRODUCTION

In mammals, two interferon inducible proteins, ADAR1 and DAI, contain Zalpha domains, which is the first protein motif that has been found to specifically recognize the high-energy conformation of double-stranded DNA known as Z-DNA. It is believed that this domain is involved in the recognition of features of foreign nucleic acids, and DAI is shown to mediate activation of the interferon genes in response to DNA or viral infection in certain cell types (1). Moreover, mutations within the Zalpha domain of the ADAR1 RNA editing enzyme, similarly to those in the ADAR1 catalytic domain, are shown to cause the Aicardi Goutières syndrome (2), an autoimmune condition. This indicates a role of ADAR1 and its Zalpha domain in nucleic acids clearance and suppression of type I interferon signaling.

Another critical protein in the interferon pathway is the RNA dependent protein kinase (PKR), which, among many other functions, mediates the shutdown of protein translation through the phosphorylation of Ser51 of the alpha subunit of the eukaryotic translation initiation factor 2 (3,4). Viral double-stranded RNA is the signal that activates PKR, and this activation is mediated by its two N-terminal dsRNA binding domains (dsRBD). To counteract PKR, Pox-viruses encode E3L a protein that contains a dsRNA-binding domain and a Zalpha domain and is known to be a potent inhibitor of the interferon response (5). The available evidence suggests that both nucleic acids interacting domains of E3L are required for full inhibition of antiviral responses (6,7) and that they compete with cellular pathogen-associated molecular pattern sensors like PKR and DAI to control their responses and evade detection.

Unexpectedly, several fish species encode a PKR like kinase in which the two dsRNA-binding domains are substituted by two Z-DNA-binding Zalpha domains (8). Although initially it appeared that this kinase (PKZ) replaces PKR in these species, genome analysis revealed that both PKR and PKZ co-exist and have complementary functions (9,10). Interestingly, a recently identified Zalpha containing protein ORF112 was found in Alloherpesviridae, which infect fish species that bear PKZ. Structural and biochemical analysis confirmed its Zalpha fold and Z-DNA-binding properties (11), suggesting a role for ORF112 as an inhibitor of PKZ.

PKZ can phosphorylate eukaryotic translation initiation factor 2 (12), although recently emerged evidence suggests that *in vivo* it may target another initiation factor (13). PKZ interacts with CpG repeats (14), the DNA sequence that is more prone to adopt the left-handed helical conformation of Z-DNA. The ability of the PKZ Zalpha domain to bind DNA CpG repeats has been demonstrated in gel-retardation experiments, and circular dichroism has shown that the bound DNA is in the left-handed helical conformation (15). The structural details of the PKZ Zalpha interactions are unknown, despite a report of crystallization of the *Carassius auratus* Zalpha^PKZ^ with nucleic acids (16).

Although the data mentioned previously suggest that PKR and PKZ respond to different molecules (dsRNA and dsDNA, respectively), this may be misleading as dsRNA has been shown to adopt a similar conformation to Z-DNA and to interact with the Zalpha domain in a manner similar to Z-DNA (17,18). Studies so far have not explored CpG repeats in RNA as target of PKZ Zalpha domains. Thus, it remains possible that the *in vivo* target of PKZ may as well be dsRNA, as it is for PKR.

The structure of the Zalpha domain and its interactions with DNA and RNA has been extensively studied. Representative co-crystal structures of Zalpha domains with DNA have been determined for ADAR1 (19), DAI (20,21) and E3L (22) defining the major interactions and critical residues for DNA binding. All the Zalpha/DNA complexes determined thus far are based on a (CG)_3_ dsDNA oligonucleotide as a ligand, which is thought to comprise the minimal binding site. Aiming to understand conformational junctions, we and others have determined two structures using longer DNA ligands. However, in these cases, the sequences do not comply with the dinucleotide repeat along their entire length, forming either a B-Z DNA junction (23) or a Z-Z DNA junction (24).

Zalpha binds Py/Pu repeats under conditions unfavorable for the Z-DNA formation, and it is the presence of Zalpha domains that drives the transition of the DNA structure to the left-handed (Z) conformation. The mechanism behind this transition is not well understood. Two alternative explanations suggest that either Zalpha binds traces of Z-DNA present at equilibrium with B-DNA, shifting the equilibrium toward the left-handed conformation (25) or that somehow Zalpha actively catalyzes the DNA conformational change (26).

Although DAI, PKZ and ADAR1 contain multiple Zalpha domains (typically two), if and how such multiple domains co-operate to bind a single nucleic acid helix has been unclear, as no relevant crystal structure has been determined. Biochemical evidence suggests that the behavior of the double domain unit, which is often called Zab, is distinct from that of the individual domains (27). Similarly, we do not know how Zalpha domains arrange themselves within longer helices than the typical (CG)_3_ binding site. The only proteins known to contain a single Zalpha domain are the viral proteins E3L and ORF112, but the finding that ORF112 forms homodimers raises the possibility that even in these cases the functional unit for DNA interaction might be the double Zalpha domain (11). Thus, there is a clear need for structural information concerning how proteins containing multiple Zalpha domains recognize Z DNA helices.

Here, we present the crystal structure of DrZalpha^PKZ^ in complex with a 12 bp CG repeat and describe novel protein/DNA interactions beyond the minimal binding site as well as the arrangement of multiple domains along the DNA duplex. We uncover extended interactions with the CG substrate that help explain the mechanism of Z-DNA binding and its subsequent stabilization by Zalpha domains.

## MATERIALS AND METHODS

### Cloning and mutagenesis

A plasmid containing the *Danio rerio* protein kinase containing Z-DNA-binding domains (DrPKZ) full length (NCBI accession number NP_001035466.1) was kindly provided by Dr Silvia Correia. This plasmid was used as a template to amplify by PCR the fragment corresponding to the first Z-DNA-binding domain (DrZalpha^PKZ^, residues Ser5-Ser70). The DrZalpha^PKZ^ was cloned in the pET28b vector in frame with an N-terminal, cleavable poly-His tag, giving an 8.4 kDa (72 aa) product after thrombin treatment. Site-directed mutants of DrZalpha^PKZ^ were prepared using NZYMutagenesis kit (Nzytech) following the manufacturer protocol.

### Expression and purification

The protein was expressed in BL21 (DE3) *Escherichia coli* cells. Cells were grown until the O.D. at 600 nm reached a value of 0.6–0.8. Expression was induced with 1 mM IPTG. After 3 h at 37°C, cells were harvested by centrifugation (6000 *g*). All chromatographic steps were performed on an AKTA purifier system (GE Healthcare). Typically, 5 g of dry cell pellet were chemically lysed with Bugbuster (Novagen), and the cleared extract was loaded on a Hitrap IMAC column (chelating Ni ions). The protein was eluted with a linear 5–500 mM imidazole gradient. The eluate was supplemented with 3 U/µl of thrombin (Novagen) to get rid of the His_6_ tag and was dialyzed o/n at 4°C against 10 mM Tris (pH 8.4), 75 mM NaCl, 1.25 mM CaCl_2_. The cleaved protein was further purified on a MonoS column, equilibrated with 20 mM HEPES (pH 7.0), 20 mM NaCl. A linear 20–600 mM NaCl gradient was used to elute DrZalphaPKZ. Finally, selected fractions were pooled and concentrated to 20–30 mg/ml in the MonoS equilibration buffer and used for crystallization experiments.

### Crystallization

Three different oligonucleotides were used in initial crystallization trials: the self-complementary T(CG)_3_ and T(CG)_6_ and the hetero-duplex T(CA)_3_/T(TG)_3_. Oligonucleotides were purchased from Integrated DNA Technologies and annealed by a linear temperature gradient (80–10°C) for 16 h. Annealed duplexes were mixed with the purified protein at protein/duplex molar ratios of 0.6:0.3 mM (7-mers) or 0.8:0.2 mM (13-mer) and incubated 30’ at 37°C. Crystallizations were initially performed with a Cartesian mini-bee robot using the sitting drop, vapor diffusion method at 20°C. In all, 0.1 µl of the protein/DNA mixtures were mixed with 0.1 µl of solutions from Structure screen 1 and 2 HT-96 (Molecular Dimensions). Crystals, mostly tiny needles, were only obtained for the complex with T(CG)_6_ duplex. After optimization, two crystal forms having rod and hexagonal morphologies could be produced under similar conditions: 1.8 M ammonium sulfate, 0.1 M sodium acetate (pH 5.4–5.6) with the presence of 5–7.5% glycerol promoting formation of the hexagonal form.

### Data collection and structure determination

Crystals were flash frozen in their reservoir solution supplemented with 20% glycerol. Complete data sets for both crystal forms were collected at European Synchrotron Radiation Facility, Grenoble on beamline ID23-1 using a Pilatus array. Both data sets were processed with iMOSFLM (28) and scaled with SCALA (29). Diffraction of the crystals extends to 2 (Hexagonal) and 1.8 Å (Tetragonal) resolutions, and the collected data have an Rmerge of 7.4 and 8.8% for the tetragonal and the hexagonal crystal forms, respectively. The two crystals not only differ in their habit but also in the geometry of the unit cells. The hexagonal crystal form belongs to the P6_1_22 space group, whereas the rod shaped crystals belong to the tetragonal space group I422 with different unit cell parameters (see Table 1). The estimated AU content suggested presence of one DNA strand and two protein molecules for the hexagonal form, whereas for the tetragonal the asymmetric unit appeared too small for the expected constituents. We successfully phased the hexagonal crystals by molecular replacement using the ADAR1 Zalpha/DNA complex (PDB code 1QBJ) as template and the program PHASER (30). In the asymmetric unit of the hexagonal crystals, two Zalpha molecules bind a single-stranded 12-mer DNA. The crystallographic 2-fold axis creates the biological unit of 12 bp duplex with four Zalpha domains. For the tetragonal crystals, a molecular replacement solution was obtained using as template a partially refined model of the hexagonal crystal comprising, however, only a single Zalpha and a (CG)_3_ DNA strand. The electron density map was of good quality, suggesting that the solution was correct and that crystallographic symmetry had to be applied twice to recreate the biological unit. Extending the analysis to the neighboring AUs shows that the DNA duplexes stack as pseudo-continuous helices along the Z-DNA axis spanning the entire crystal. Refinement for both structures was performed with PHENIX (31) and manual model building with COOT (32). Data collection, phasing and refinement statistics of the two structures are listed in Table 1. Nucleic acids structures were analyzed with 3DNA (33), whereas PISA (34) was used for the definition of the interacting surfaces. Structural alignments of Zalpha domains were performed with PDBeFold (35). Both structures and reflection files have been deposited to RCSB with PDB ID code 4LB6 for the tetragonal and PDB ID code 4LB5 for the hexagonal crystal form.

**Table 1.** Data collection and refinement statistics

	Hexagonal		Tetragonal
Data collection			
Space group	P6_1_22		I422
Cell dimensions:			
a, b, c (Å)	56.9, 56.9, 218.8		110.9, 110.9, 43.1
α, β, γ (°)	90, 90, 120		90, 90, 90
Wavelength (Å)	0.97		0.97
Total reflections	109 200		56 184
Unique reflections	15 026		12 161
Resolution range (Å)	40.81–2.00 (2.11–2.00)^a^		32.55–1.80 (1.90–1.80)
R_merge_ (%)	8.8 (53.0)^a^		7.4 (67.9)
I/σ(I)	13.3 (3.7)^a^		11.3 (2.2)
Completeness (%)	99.6 (100)^a^		96.4 (96.0)
Multiplicity	7.3 (7.2)^a^		4.6 (4.7)
Refinement	
Resolution range (Å)	29.3–2.0		28.1–1.8
R_work_/R_free_^b^ (%)	18.78/22.34		18.09/21.16
RMSD			
Bonds (Å)	0.006		0.019
Angles (°)	1.074		2.062
Ramachandran plot			
In preferred regions (%)	99.1		98.4
In allowed regions (%)	0.9		1.6
Outliers (%)	0		0
Mean B-factors (Å^2^)	49.1		36.6
Protein	53.0		39.8
DNA	34.2		20.6
Solvent	45.6		40.7

^a^Numbers in parentheses represent values in the highest resolution shell.

^b^R_work_/R_free_:Σ||F_obs_| − |F_calc_||/Σ|Fobs| where F_obs_ and F_calc_ are observed and calculated structure factors respectively.

### DNA-binding assays

For the characterization of the DNA-binding activity both gel mobility shift and circular dichroism (CD) spectroscopy were used. In gel-shift experiments, 25 µM of the same duplexes used for crystallization were mixed with increasing concentrations of protein (covering 0:1–6:1 molar ratios) in a total volume of 10 µl. After 30′ incubation at 37°C, samples were run on a 6% DNA retardation gel (Invitrogen). Gels were stained with RedSafe (iNtRON biotechnology) followed by coomassie blue to visualize both nucleic acids and proteins. CD experiments were performed on a Jasco J-815 CD instrument, using a 200 µl, 0.1 mm path-length cuvette. Samples were prepared as for the gel-shift experiments, but higher concentrations of nucleic acids were used (40–50 µM). Spectra were recorded between 240 and 320 nm as no contribution of the protein is detectable in this range.

## RESULTS

**Figure 1.**
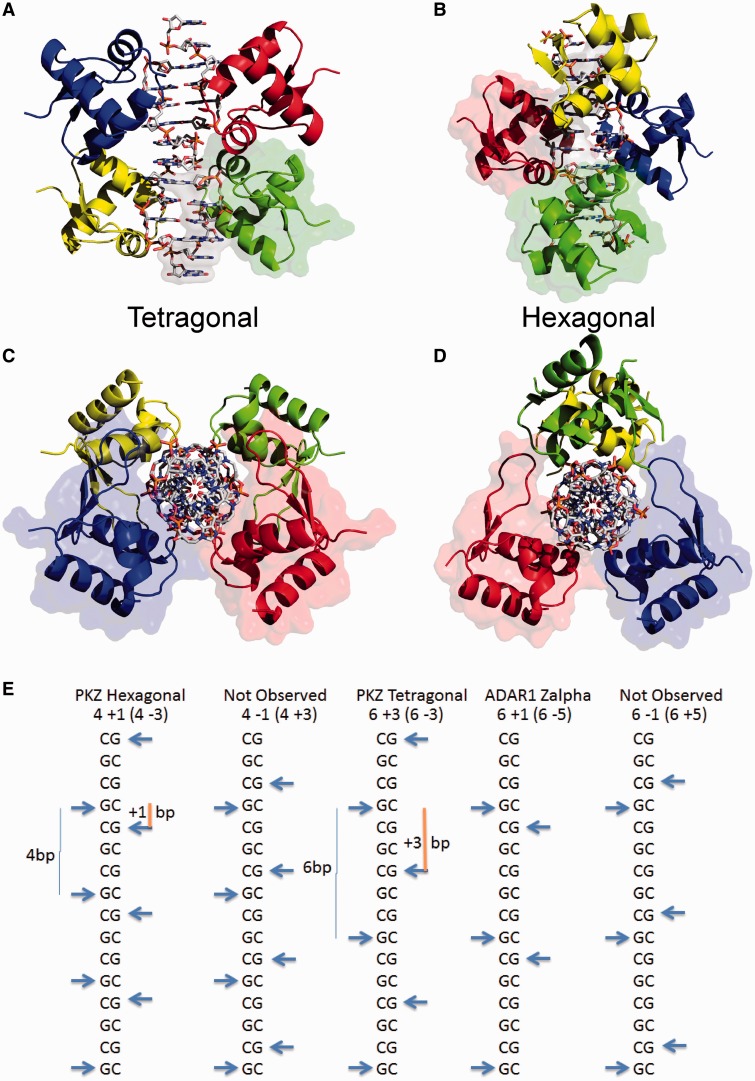
Overview of the DrZalpha^PKZ^ structures in complex with T(CG)_6_ DNA duplex and Zalpha domain organization on CG stretches. (**A–D**) Reconstructed biological assemblies for the tetragonal (A) and hexagonal (B) crystal forms seen with the DNA axis either perpendicular or along (C–D) the view line. In both structures, one DNA duplex (represented in ball-and-stick) binds 4 Zalpha molecules (cartoon). Surface representation is used to mark the content of the asymmetric unit (AU) for A and B, whereas the C and D to depict topologically close monomers wrapping the Z-DNA helix with their wings. To reconstitute the biological unit of the hexagonal crystal, the AU contents are rotated around a crystallographic 2-fold axis; twice such an operation is needed for the tetragonal crystal. (**E**) Diagram of the arrangement of the Zalpha domains along the DNA helix. The guanosines involved in the CH–π interaction with Tyr45 are used as reference and are marked by arrows. The numbers under the title indicate the binding site spacing and the relative position of binding on the other strand. For example, the model 4 + 1 indicates Zalpha binding every 4 bp on one strand and Zalpha binding on the next base pair on the second strand. In parenthesis is shown a symmetric equivalent arrangement. Included here are the two structures described, the prototypic ADAR1 Zalpha and two potential arrangements that have not been observed in a Zalpha structure. Among them, only the tetragonal form (6 + 3) can propagate infinitely without leading to protein–protein clashes (see also Supplementary Figure S1).

We expressed and purified the PKZ Zalpha domain (DrZalpha^PKZ^) encompassing residues 5–70 of zebrafish PKZ (DrPKZ). The protein was crystallized with an oligonucleotide forming a self-complementary (CG)_6_ DNA duplex with a single T base overhang and resulted in crystals belonging to two different space-groups growing under similar conditions. The determination of both structures using molecular replacement (see Table 1) revealed that the two crystal forms represent two different arrangements of protein molecules on the DNA helix due to the repeated nature of the DNA sequence. In the form that belongs to the tetragonal space-group I422 the two binding sites on the same DNA molecule are related with crystallographic symmetry resulting in the presence of just half the DNA segment in the asymmetric unit and the appearance of a continuous DNA helix throughout the crystal lattice (Figure 1A and C). The asymmetric unit of the other (hexagonal) crystal form, which belongs to the P6_1_22 space group, contains a single 12 bp-long DNA strand and two DrZalpha^PKZ^ monomers. Overall, the two structures are superimposable in regard to their primary interactions with the nucleic acid and they mainly differ for the distribution of Zalpha molecules along the duplex. Throughout the article, the hexagonal structure is used as reference, although, if not otherwise stated, descriptions apply to both structures.

### Overview of the structures

The differential arrangement of the Zalpha domains in the two crystal forms is depicted in Figure 1A–D. The tetragonal form shows a 6 bp distance between Zalpha binding sites along a single DNA strand, as defined by the equivalent CH-π interaction of Tyr45 with a G in *syn* conformation. On the other hand, the hexagonal form shows a 4 bp distance between binding sites with two monomers contacting the central part of the DNA helix and two interacting with the duplex edges (Figure 1B). In Figure 1E, we describe these arrangements in a schematic view along with other possible arrangements including the arrangement deduced from the ADAR1 Zalpha domain structure. For description purposes, we devised a nomenclature in which the first number indicates the distance between binding sites on the reference strand while the second indicates the relative position of Zalpha binding on the second strand. Modeling of the alternative Zalpha arrangements on a long Z-DNA duplex shows that the 6 + 3 arrangement observed in the tetragonal form is the only that can propagate along a continuous helix without leading to protein-protein steric clashes as seen in the crystal structure (Supplementary Figure S1).

Despite the differences in arrangement both structures reveal a Zalpha domain with all the characteristic features of the winged-Helix-Turn-Helix fold known for Zalpha domains. Little variation could be observed between the different Zalpha domains in the structure. Superposition of DrZalpha^PKZ^ with the prototypic ADAR1 Zalpha domain (PDB code 1QBJA) results in a structural alignment with an RMSD 0.520 Å^2^ (195 backbone atoms) showing no major differences between the two proteins (Supplementary Figure S2). However, a distinguishing feature of DrZalpha^PKZ^ when compared with other members of the Zalpha family is the significantly longer wing, which, along with the unique domain arrangements seen in our structure, leads to a wrapping of the protein around the DNA helix (Figure 1C and D, Supplementary Figure S2). This extended wing region contributes with additional interactions to the DNA backbone described later in the text. The DNA in both structures has a typical Z-DNA configuration similar to that in other Zalpha/DNA complexes. However, as the DNA in our complexes is longer and comprises multiple binding sites, some monomers interact with the central part of the helix and others close to the DNA edges, and this differential environment creates important differences.

### Protein–DNA interactions

**Figure 2.**
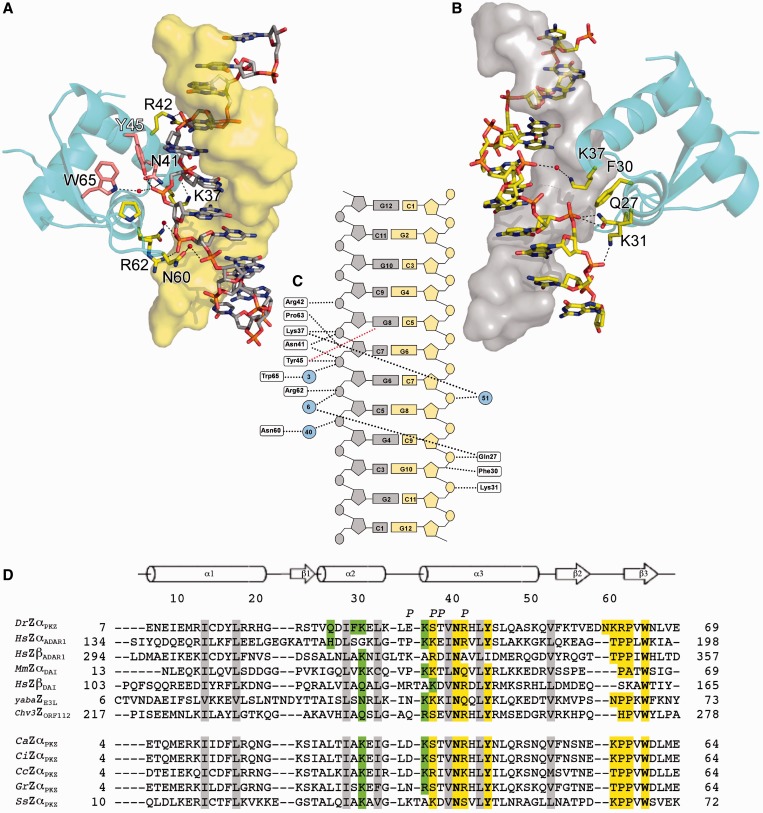
Protein contacts to the DNA. DNA contacts of PKZ Zalpha monomer B (hexagonal crystal). (**A**) Contacts with the primary DNA strand (secondary strand shown as yellow surface). (**B**) Secondary contacts with the other strand (primary strand shown as gray surface). Figure 2B is obtained by 180° rotation of Figure 2A around the DNA axis. Side chains of residues involved in DNA interactions are shown in stick representation and the conserved triad Tyr, Asn, Trp in Figure 2A is colored purple. (**C**) Schematic representation of the main protein/DNA interactions, black dotted lines are used to mark both H-bonds and van der Waals interactions, a red line indicates instead the characteristic CH-π interaction between the tyrosine and the guanosine in *syn* conformation. Cyan circles represent ordered water molecules and are numbered as in the pdb file. The coloring of the bases for the two strands is in accordance to Figure 2A and B. (**D**) Structure based alignment of DrZalpha^PKZ^ with Zalpha domains of ADAR1 (UniProt ID: P55265), DAI (UniProt ID: Q9QY24, Q9H171), E3L (UniProt ID: Q9DHS8) and ORF112 (UniProt ID: A4FTK7) and Zalpha domains of PKZ in other fish species *(Carassious auratus (Ca;* UniProt ID: Q7T2M9*)*, *Ctenopharyngodon idella (Ci;* UniProt ID: D2Y3U3*)*, *Cyprinus carpio (Cc; gb:*CF662905.2*)*, *Gobiocypris rarus (Gr;* UniProt ID: B4X9W1*)*, *Salmon salar* [Ss; UniProt ID: Q0H740)]. Highlighted are residues participating in primary (yellow) and secondary (green) interactions with DNA. Gray shade indicates hydrophobic core residues. Finally, P marks residues seen involved in protein–protein contacts.

Both structures reveal a similar pattern of protein/DNA interactions (Figure 2A–C), which can be divided into two categories. The first category involves interactions with the DNA strand facing Zalpha and includes conserved interactions described in previous Zalpha/DNA complex structures (Figure 2A). The second category comprises interactions with DNA not previously uncovered as they either extend beyond the (CG)_3_ binding site or they are disturbed by DNA edge distortions in a (CG)_3_ complex. They are however clearly visible in the (CG)_6_ complex (Figure 2B). The understanding of these interactions is facilitated by the fact that some monomers bind at the edge of the helix, as in the (CG)_3_ complexes, and others bind to the center of the helix, which we can directly compare.

The conserved triplet Tyr45, Asn41 and Trp65 interactions (Figure 2D) as well as that of Arg42 all belong to the class of primary interactions, which form critical contacts with the backbone phosphates of DNA (Figure 2C). Tyr45, as in previous structures, also contributes to the specificity through a CH-π interaction with a guanosine in the Z-DNA specific *syn* conformation, whereas Trp65 contributes with a highly conserved water-mediated interaction with the DNA phosphate oxygen. The Arg62 of the extended wing of monomer B of DrZalpha^PKZ^ forms an additional interaction with the backbone of G6 of the same strand. Interestingly, although the wings of the monomers that face the center of the DNA are well ordered, the wings of the two monomers that face the edge of the duplex are not visible in the electron density, missing residues Glu57-Asn60 and losing the Arg62 contact to DNA. Thus, these interactions are only visible in a 12 bp DNA (the one we used here) instead of the typical 6 bp binding site. All the aforementioned interactions and all known conserved interactions seen in previously determined Zalpha structures are with a single DNA strand.

### Extended protein–DNA interactions reveal contacts with both DNA strands

Although some contacts with the second DNA strand are visible in previous structures (e.g. Lys138 in Zbeta DAI), those have not been observed consistently and thus have not been considered to be part of the DNA recognition mechanism. Our structure reveals interactions with the second DNA strand involving Gln27, Phe30 and Lys31, whereas Lys37 is positioned in the middle of the minor groove bridging between the two strands (Figure 2B). Also in this case, the monomers positioned at the edge of the DNA are missing these interactions. Thus, our structure reveals multiple contacts with DNA that would be absent in a complex with (CG)_3_ DNA and suggest that the interactions of other Zalpha domains should be revisited in the view of an extended binding site.

### Protein–protein interactions

Binding to a longer DNA helix leads not only to additional protein–DNA interactions but also changes the phasing by which Zalpha domains contact DNA and consequently their relative positioning. In the prototypic Zalpha complex, the two monomers are located on opposite sides of the DNA helix with no contacts between them. In our structure, the centrally positioned monomers symmetrically contact each other (Figure 2C and D). The interaction involves the N-terminal edge of α3 of the two monomers and consists of two symmetric salt bridges between Glu36 and Arg42 and hydrogen bonds involving Thr39, Glu36 and Ser38 (Supplementary Figure S3). The guanidinium group of Arg42 is in an interesting arrangement as its Nε also forms a hydrogen bond with the DNA backbone phosphate oxygen. This interaction locks the complex from the minor groove side.

### Second strand interacting residues are critical for stable binding of Zalpha to DNA

**Figure 3.**
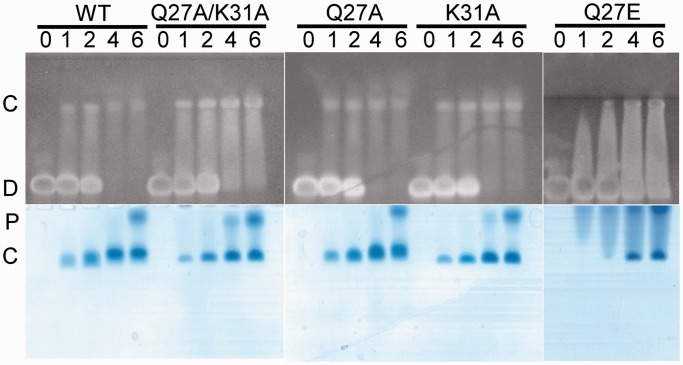
DNA binding of DrZalpha^PKZ^ mutants involved in second strand interactions. Gel-shift assay to test the binding of PKZ Zalpha and its mutants to a (CG)_7_ DNA duplex. The same polyacrylamide gel was stained for nucleic acids with RedSafe (top) and proteins with Coomassie Blue (bottom). Shown are the DNA alone (0) and band-shifts of *Dr*Zalpha^PKZ^ (WT), mutants Q27A, Q27E, K31A and the double mutant Q27A/K31A. Each protein/DNA shift is shown for four protein/DNA-duplex molar ratios: 1:1, 2:1, 4:1 and 6:1 as indicated on the top of each lane. The approximate position for free DNA (D), protein/DNA complex (C) and the free protein (P) are indicated. In all experiments, 0.25 nmoles of DNA were incubated with the indicated multiple amounts of protein before loading to the gel.

To understand the significance of the observed interactions with the second DNA strand, we created the corresponding mutants and analyzed their ability to interact with DNA *in vitro*. Several residues and ordered water molecules contribute to the formation of this relatively small (136 Å^2^) interface; amongst these, we identified Gln27 and Lys31 as main contributors. To examine the role of these residues, we produced the following mutants: Gln27Ala, Lys31Ala and the double mutant Gln27Ala/Lys31Ala plus the Gln27Glu, which introduces a strong negative charge expected to repel the oxygen atoms of the DNA phosphate groups. All the mutant proteins were well expressed and stable, suggesting that they did not affect protein folding. We tested all the mutant proteins for their ability to bind a T(CG)_7_ duplex in gel-shift experiments (Figure 3). The Gln27Ala mutation alone had a minimal or no effect on DNA binding, and Lys31Ala shows only a mild loss of affinity as evident by an increase in free protein. The effect of the combined mutations however, was stronger, as free protein is evident even in the 4:1 ratio, suggesting a synergetic effect of the mutations. In contrast to the weak effects of Gln27Ala and Lys31Ala, we observed strong loss of DNA-binding for the Gln27Glu mutant. This highlights the close interaction of this residue with the phosphate oxygen atoms, confirming that this contact is formed and is critical in solution.

## DISCUSSION

Here, we described the first crystal structure of a PKZ DNA/RNA binding Zalpha domain. In addition to revealing the characteristics of the DrZalpha^PKZ^ protein domain, this is the first structure of a Zalpha domain where the CpG repeats in the bound DNA extend beyond the minimal (CG)_3_ binding site without interruption of the dinucleotide repeat. Thus, it enables us to better determine the binding mode of these domains in the context of a naturally long DNA duplex. Beyond the fine details of DrZalpha^PKZ^/DNA contacts, the two structures address two additional questions: what are the possible arrangements of multiple Zalpha domains on a long DNA duplex and if the presumed minimal binding site contains all the elements of protein–DNA interactions that occur with a long DNA duplex.

The principal interactions seen between DrZalpha^PKZ^ and the DNA helix closely resemble what is known from other Zalpha domains, with the critical amino acids not only conserved but maintaining essentially identical conformations. Additional interactions arise from the presence of an extended DNA duplex used in the present study and the question is to which extent these interactions are PKZ specific, or alternatively they represent features of Zalpha domains that have not been seen in the previous structures due to deformations of the DNA edges. DrZalpha^PKZ^ is characterized by an extended by 2aa wing, which includes Arg62, a residue that interacts with the primary DNA strand. This interaction is lost for monomers that bind at the edge of the DNA duplex along with the structure of the entire wing. This interaction is apparently a unique feature of DrZalpha^PKZ^ as most Zalpha domains have a Pro residue at the structurally equivalent position.

Use of the extended DNA substrate revealed unanticipated interactions with the second strand and insights into how these interactions are lost at the edge of the DNA helices. This latter observation clearly shows that distortions of Z-DNA at the edges of the duplex disturb the Zalpha/DNA contacts. If these interactions are important then how do Zalpha domains strongly bind (CG)_3_ oligonucleotides? One answer to this question is that at least the interaction through Gln27, which contacts the last phosphate does happen with (CG)_3_ in solution but is lost only in the crystal, as crystal packing distorts the DNA duplex. This is supported from the fact that His159 in Zalpha^ADAR1^ which occupies the equivalent to Gln27 position is in position to contact DNA, but it assumes alternative conformations of which the dominant is not in contact with DNA (19,24). In addition, the effects of second strand interactions may reveal themselves only when protein and DNA concentrations are low, and the intermediate complex of a single Zalpha with DNA has to be sustained for longer periods. Among the residues involved in the binding of the secondary strand, Gln27 and Lys31 seem the most important, as they both interact through a direct hydrogen bond to phosphates of the DNA backbone. Lys31 is well conserved among Zalpha domains, although occasionally substituted by arginine, asparagine or glutamine (Figure 2D). Gln27 on the other hand shows a low degree of conservation, even within PKZs, and often a hydrophobic residue can be found in this position. Only Zalpha^ADAR1^ seems to share this interaction having His159 at an equivalent position. *In vitro* the Lys31Ala mutant shows a slightly stronger effect in binding compared to Gln27Ala and only replacing this residue with glutamic acid makes its important position evident. Overall, the second strand interactions do not show the same extent of conservation seen for the primary interactions, but other amino acid changes in different Zalpha domains seem to compensate, offering alternative second strand interactions. Thus, these interactions in the final protein/DNA complex seem to be supportive, and they lack the specificity of the primary strand interactions; they may however play an important role in transient binding–intermediate interactions.

Although, as previously mentioned, different theories on the Zalpha-dependent formation of Z-DNA exist, they all agree on the obligatory step of the formation of an intermediate where only one strand of the already Z-DNA duplex is bound to a Zalpha domain. How a single Zalpha domain can maintain the unstable helix in the left-handed conformation long enough for a second monomer to arrive is unclear, particularly at low protein concentrations. The second strand interactions observed here may play a key role. This locking of the DNA structure may have no great significance at the high concentrations of protein and DNA used in our, and others *in vitro* assays, but this locking maybe crucial when the Zalpha concentrations are low. This also might provide an explanation why all cellular proteins containing Zalpha domains bear multiple domains as likely the second domain is used to confer further stabilization by forming primary interactions with the second strand. The single domain viral proteins E3L and ORF112 might achieve the same by forming dimers as suggested by the ORF112 structure (11). The configurations observed in the two structures of DrZalpha^PKZ^ offer critical insights in this regard.

**Figure 4.**
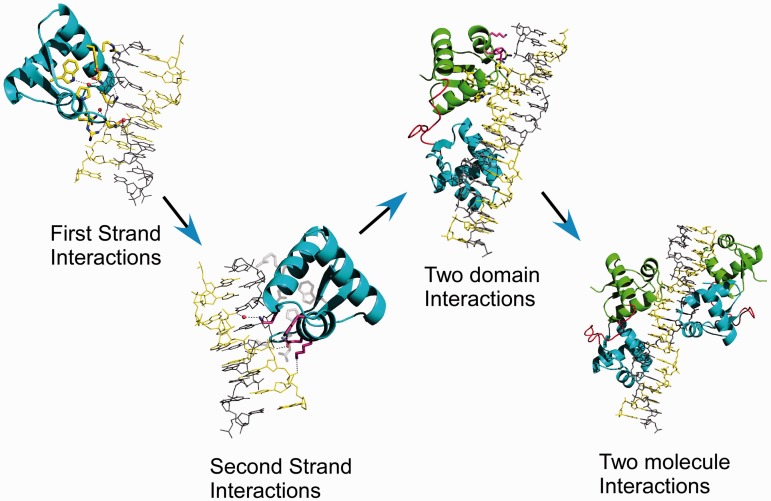
Model of the mechanism of stabilization of the Z-DNA conformation by Zalpha domains. Interactions of Zalpha with a single strand allow recognition of the DNA conformation followed by interactions with the second strand that transiently locks the DNA in left-handed form. A second monomer from the same protein interacts with the second strand forming a stable complex. Binding of a second protein with two Zalpha domains will extend the left-handed helix to three binding sites or 18 bp.

The two crystal forms of DrZalpha^PKZ^ with the (CG)_6_ oligonucleotide provide a range of alternative arrangements of Zalpha domains in a helix longer than the minimal binding site. In both cases, four monomers are bound on the 12 bp duplex in agreement with the stoichiometry of the interaction deduced from gel-shift experiments where a protein/DNA ratio 4:1 is required for the complete shift (Figure 3, WT). Based on the two structures, we created models of all possible arrangements, and we found that the tetragonal structure denoted as 6 + 3 (Figure 1E) represents the only arrangement of Zalpha domains that can propagate uniformly along a DNA helix without leading to protein–protein clashes (Supplementary Figure S2). Although other arrangements may occur, as we see in different Zalpha crystal structures, the tetragonal arrangement is expected to settle at equilibrium with a long DNA helix. Based on this arrangement, we evaluated by modeling which of the possible pairs of Zalpha domains could represent the double Zalpha domain of PKZ, DAI and ADAR1, by being positioned in a way that they could satisfy constrains of a C-Terminus – N-terminus linkage between domains. We found that among the possible pairs one is topologically preferable, as it can accommodate a non-structured but geometrically possible poly-alanine linker, 15 residues long (Figure 4, two domain interactions). DAI is the only protein for which we have concrete experimental evidence that both Zalpha domains bind Z-DNA. In the DAI case, the actual linker between the two domains is generally not conserved and the length varies, ranging from the 21 aa length of the hamster protein to the 52 aa of the common cattle and with the shorter linker in agreement with our minimal length estimation. Although, the described arrangement is the most plausible, longer linkers may allow alternative configurations, as it is suggested from the fact that for DAI alternative splicing creates protein variants with different linker lengths with potential functional consequences.

### A revised model for the recognition of Z-DNA by Zalpha domains

Figure 4 summarizes a model for the interaction of proteins with multiple Zalpha domains with DNA/RNA based on the findings of the structures described here. The model suggests successive steps of stabilization of the Z-DNA conformation and predicts propagation of the left-handed conformation from a starting nucleation point. The transition starts with the highly conserved primary recognition interactions of a Zalpha with a single strand of Z-DNA. Once recognition occurs, the secondary interactions with the second strand lock DNA in the left-handed conformation for as long as needed for the second Zalpha domain of the same protein to form primary interactions with the other strand. Binding of the second Zalpha domain (Zbeta) would form the primary stable complex and suggests that the natural target DNA involves at least two binding sites or at least the 12 bp observed in the structure presented here. Once the stable complex is formed, a second protein can contribute the next two Zalpha domains, which however would increase the length of the interacting DNA region to three binding sites or 18 bp. A long perfect alternating Purine/Pyrimidine sequence is expected to result in an aggregate of proteins on DNA, something that has previously been observed for the RNA sensor MDA5 (36). Although such long perfect dinucleotide repeats are not frequent in natural sequences, negative supercoiling has been shown to be able to drive imperfect repeats in the left-handed conformation and Zalpha domains are capable to interact with them (37). Transcription is a known source of negative supercoiling *in vivo* but normally occurs in the nucleus, whereas the localization of the proteins that contain Zalpha domains is primarily cytoplasmic. The source of negative supercoiling in the cytoplasm remains to be uncovered.

The ADAR1 Zbeta domain has been shown to be unable to interact with nucleic acids on its own (38), and thus it appears that ADAR1 binding to DNA does not rely on the mechanism proposed here. However, it is likely that the second domain to interact would not need such a high affinity, and it would be sufficient to provide non-specific support interactions that could lock the complex in the left-handed conformation. Indeed, experiments show that although Zbeta forms no complex with DNA alone, it is still capable of altering the behavior of Zalpha when present in the context of a Zab construct (27,39). Alternatively, it has been shown that ADAR1 functions as a dimer (40), in which case each monomer might contribute a Zalpha for the interaction in an arrangement likely different from the model described here.

Overall, the structural analysis of DrZalpha^PKZ^ provides a first insight into the higher-level organization of Zalpha domains on DNA applicable to other Zalpha containing proteins. This higher-order organization is likely a prerequisite for the downstream activity of these innate immunity sensor proteins. Finally, it is worth to point out that the pair of structures of DrZalpha^PKZ^ presented here and the previously determined of ORF112 from the Cyprinid Herpesvirus 3 (11) could provide important tools for the study of the host—pathogen competition in economically important fish species.

## ACCESSION NUMBERS

PDB ID 4LB6, PDB ID 4LB5.

## SUPPLEMENTARY DATA

Supplementary Data are available at NAR Online.

## FUNDING

Fundação para a Ciência e a Tecnologia (FCT) [PTDC/BIA-PRO/112962/2009 to A.A., SFRH/BPD/71629/2010 to M.deR.]; European Commission Marie Curie IRG Grant [PIRG03-GA-2008-231000 to A.A.]; Data collection was supported by the ESRF BAG program [MX1291]. Funding for open access charge: FCT/Instituto Gulbenkian de Ciência.

*Conflict of interest statement*. None declared.

## Supplementary Material

Supplementary Data

## References

[1] Takaoka A, Wang Z, Choi MK, Yanai H, Negishi H, Ban T, Lu Y, Miyagishi M, Kodama T, Honda K (2007). DAI (DLM-1/ZBP1) is a cytosolic DNA sensor and an activator of innate immune response. Nature.

[2] Rice GI, Kasher PR, Forte GM, Mannion NM, Greenwood SM, Szynkiewicz M, Dickerson JE, Bhaskar SS, Zampini M, Briggs TA (2012). Mutations in ADAR1 cause Aicardi-Goutieres syndrome associated with a type I interferon signature. Nat. Genet..

[3] Munir M, Berg M (2013). The multiple faces of proteinkinase R in antiviral defense. Virulence.

[4] Metz DH, Esteban M (1972). Interferon inhibits viral protein synthesis in L cells infected with vaccinia virus. Nature.

[5] Haig DM, McInnes CJ, Thomson J, Wood A, Bunyan K, Mercer A (1998). The orf virus OV20.0L gene product is involved in interferon resistance and inhibits an interferon-inducible, double-stranded RNA-dependent kinase. Immunology.

[6] Langland JO, Jacobs BL (2004). Inhibition of PKR by vaccinia virus: role of the N- and C-terminal domains of E3L. Virology.

[7] Kim YG, Muralinath M, Brandt T, Pearcy M, Hauns K, Lowenhaupt K, Jacobs BL, Rich A (2003). A role for Z-DNA binding in vaccinia virus pathogenesis. Proc. Natl Acad. Sci. USA.

[8] Rothenburg S, Deigendesch N, Dittmar K, Koch-Nolte F, Haag F, Lowenhaupt K, Rich A (2005). A PKR-like eukaryotic initiation factor 2alpha kinase from zebrafish contains Z-DNA binding domains instead of dsRNA binding domains. Proc. Natl Acad. Sci. USA.

[9] Liu TK, Zhang YB, Liu Y, Sun F, Gui JF (2011). Cooperative roles of fish protein kinase containing Z-DNA binding domains and double-stranded RNA-dependent protein kinase in interferon-mediated antiviral response. J. Virol..

[10] Rothenburg S, Deigendesch N, Dey M, Dever TE, Tazi L (2008). Double-stranded RNA-activated protein kinase PKR of fishes and amphibians: varying the number of double-stranded RNA binding domains and lineage-specific duplications. BMC Biol..

[11] Tome AR, Kus K, Correia S, Paulo LM, Zacarias S, de Rosa M, Figueiredo D, Parkhouse RM, Athanasiadis A (2013). Crystal structure of a poxvirus-like zalpha domain from cyprinid herpesvirus 3. J. Virol..

[12] Bergan V, Jagus R, Lauksund S, Kileng O, Robertsen B (2008). The Atlantic salmon Z-DNA binding protein kinase phosphorylates translation initiation factor 2 alpha and constitutes a unique orthologue to the mammalian dsRNA-activated protein kinase R. FEBS J..

[13] Taghavi N, Samuel CE (2013). RNA-dependent protein kinase PKR and the Z-DNA binding orthologue PKZ differ in their capacity to mediate initiation factor eIF2alpha-dependent inhibition of protein synthesis and virus-induced stress granule formation. Virology.

[14] Wu CX, Wang SJ, Lin G, Hu CY (2010). The Zalpha domain of PKZ from Carassius auratus can bind to d(GC)(n) in negative supercoils. Fish Shellfish Immunol..

[15] Lu P, Deng S, Zhu Y, Yan Y, Liu Y, Hu C (2012). The Zalpha domain of fish PKZ facilitates the B-Z conformational transition of oligonucleotide DNAs with d(GC)(n) inserts. Acta Biochim. Biophys. Sin. (Shanghai).

[16] Kim D, Hwang HY, Kim YG, Kim KK (2009). Crystallization and preliminary X-ray crystallographic studies of the Z-DNA-binding domain of a PKR-like kinase (PKZ) in complex with Z-DNA. Acta Crystallogr. Sect. F Struct. Biol. Cryst. Commun..

[17] Brown BA, Lowenhaupt K, Wilbert CM, Hanlon EB, Rich A (2000). The zalpha domain of the editing enzyme dsRNA adenosine deaminase binds left-handed Z-RNA as well as Z-DNA. Proc. Natl Acad. Sci. USA.

[18] Placido D, Brown BA, Lowenhaupt K, Rich A, Athanasiadis A (2007). A left-handed RNA double helix bound by the Z alpha domain of the RNA-editing enzyme ADAR1. Structure.

[19] Schwartz T, Rould MA, Lowenhaupt K, Herbert A, Rich A (1999). Crystal structure of the Zalpha domain of the human editing enzyme ADAR1 bound to left-handed Z-DNA. Science.

[20] Ha SC, Kim D, Hwang HY, Rich A, Kim YG, Kim KK (2008). The crystal structure of the second Z-DNA binding domain of human DAI (ZBP1) in complex with Z-DNA reveals an unusual binding mode to Z-DNA. Proc. Natl Acad. Sci. USA.

[21] Schwartz T, Behlke J, Lowenhaupt K, Heinemann U, Rich A (2001). Structure of the DLM-1-Z-DNA complex reveals a conserved family of Z-DNA-binding proteins. Nat. Struct. Biol..

[22] Ha SC, Lokanath NK, Van Quyen D, Wu CA, Lowenhaupt K, Rich A, Kim YG, Kim KK (2004). A poxvirus protein forms a complex with left-handed Z-DNA: crystal structure of a Yatapoxvirus Zalpha bound to DNA. Proc. Natl Acad. Sci. USA.

[23] Ha SC, Lowenhaupt K, Rich A, Kim YG, Kim KK (2005). Crystal structure of a junction between B-DNA and Z-DNA reveals two extruded bases. Nature.

[24] de Rosa M, de Sanctis D, Rosario AL, Archer M, Rich A, Athanasiadis A, Carrondo MA (2010). Crystal structure of a junction between two Z-DNA helices. Proc. Natl Acad. Sci. USA.

[25] Bae S, Kim D, Kim KK, Kim YG, Hohng S (2011). Intrinsic Z–DNA is stabilized by the conformational selection mechanism of Z-DNA-binding proteins. J. Am. Chem. Soc..

[26] Kang YM, Bang J, Lee EH, Ahn HC, Seo YJ, Kim KK, Kim YG, Choi BS, Lee JH (2009). NMR spectroscopic elucidation of the B-Z transition of a DNA double helix induced by the Z alpha domain of human ADAR1. J. Am. Chem. Soc..

[27] Schwartz T, Lowenhaupt K, Kim YG, Li L, Brown BA, Herbert A, Rich A (1999). Proteolytic dissection of Zab, the Z-DNA-binding domain of human ADAR1. J. Biol. Chem..

[28] Leslie AGW, Powewll HR (2007). Evolving Methods for Macromolecular Crystallography.

[29] Kabsch W (2010). Xds. Acta Crystallogr. D Biol. Crystallogr..

[30] McCoy AJ, Grosse-Kunstleve RW, Adams PD, Winn MD, Storoni LC, Read RJ (2007). Phaser crystallographic software. J. Appl. Crystallogr..

[31] Adams PD, Afonine PV, Bunkoczi G, Chen VB, Davis IW, Echols N, Headd JJ, Hung LW, Kapral GJ, Grosse-Kunstleve RW (2010). PHENIX: a comprehensive Python-based system for macromolecular structure solution. Acta Crystallogr. D Biol. Crystallogr..

[32] Emsley P, Lohkamp B, Scott WG, Cowtan K (2010). Features and development of Coot. Acta Crystallogr. D Biol. Crystallogr..

[33] Lu XJ, Olson WK (2003). 3DNA: a software package for the analysis, rebuilding and visualization of three-dimensional nucleic acid structures. Nucleic Acids Res..

[34] Krissinel E, Henrick K (2007). Inference of macromolecular assemblies from crystalline state. J. Mol. Biol..

[35] Krissinel E, Henrick K (2004). Secondary-structure matching (SSM), a new tool for fast protein structure alignment in three dimensions. Acta Crystallogr. D Biol. Crystallogr..

[36] Peisley A, Jo MH, Lin C, Wu B, Orme-Johnson M, Walz T, Hohng S, Hur S (2012). Kinetic mechanism for viral dsRNA length discrimination by MDA5 filaments. Proc. Natl Acad. Sci. USA.

[37] Lushnikov AY, Brown BA, Oussatcheva EA, Potaman VN, Sinden RR, Lyubchenko YL (2004). Interaction of the Zalpha domain of human ADAR1 with a negatively supercoiled plasmid visualized by atomic force microscopy. Nucleic Acids Res..

[38] Athanasiadis A, Placido D, Maas S, Brown BA, Lowenhaupt K, Rich A (2005). The crystal structure of the Zbeta domain of the RNA-editing enzyme ADAR1 reveals distinct conserved surfaces among Z-domains. J. Mol. Biol..

[39] Kim YG, Lowenhaupt K, Schwartz T, Rich A (1999). The interaction between Z-DNA and the Zab domain of double-stranded RNA adenosine deaminase characterized using fusion nucleases. J. Biol. Chem..

[40] Cho DS, Yang W, Lee JT, Shiekhattar R, Murray JM, Nishikura K (2003). Requirement of dimerization for RNA editing activity of adenosine deaminases acting on RNA. J. Biol. Chem..

