# Supplementary material for: Structural basis for Z-DNA binding and stabilization by the zebrafish Z-DNA dependent protein kinase PKZ
Source: Nucleic Acids Res. 2013 Aug 23;41(21):9924–33. doi: 10.1093/nar/gkt743 (PMC3834819; doi:10.1093/nar/gkt743)
Supplement: Supplementary Data [file supp_41_21_9924__index.html]

Structural basis for Z-DNA binding and stabilization by the zebrafish Z-DNA dependent protein kinase PKZ — Structural basis for Z-DNA binding and stabilization by the zebrafish Z-DNA dependent protein kinase PKZ — Supplementary Data 

# Structural basis for Z-DNA binding and stabilization by the zebrafish Z-DNA dependent protein kinase PKZ

## Supplementary Data

files

**Files in this Data Supplement:**

- Supplementary Data - zip file
