# Supplementary material for: Structural basis for Z-DNA binding and stabilization by the zebrafish Z-DNA dependent protein kinase PKZ
Source: Nucleic Acids Res. 2013 Aug 23;41(21):9924–33. doi: 10.1093/nar/gkt743 (PMC3834819; doi:10.1093/nar/gkt743)
Supplement: Supplementary Data [file supp_gkt743_suppl_data.zip › nar-01793-f-2013-File009.zip!/4LB6/new-entry_04.pdf]

PROCHECK

Main-chain parameters

new-entry

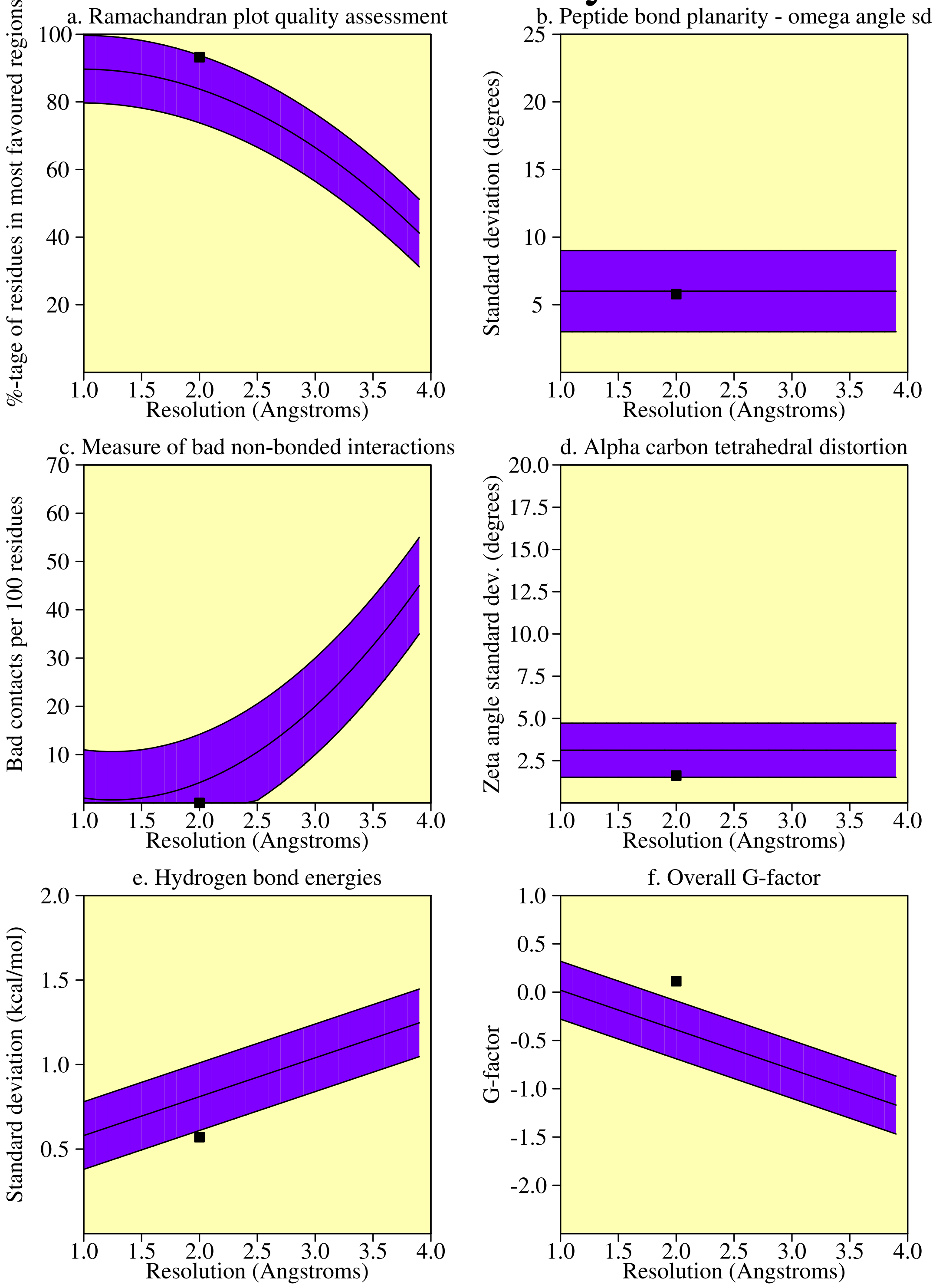

Plot statistics

Stereochemical parameter	No. of data pts	Parameter value	Comparison values Typical value	Band width	No. of band widths from mean	
a. %-tage residues in A, B, L	59	93.2	83.8	10.0	0.9	Inside
b. Omega angle st dev	62	5.8	6.0	3.0	-0.1	Inside
c. Bad contacts / 100 residues	0	0.0	4.2	10.0	-0.4	Inside
d. Zeta angle st dev	62	1.6	3.1	1.6	-0.9	Inside
e. H-bond energy st dev	41	0.6	0.8	0.2	-1.2	BETTER
f. Overall G-factor	63	0.1	-0.4	0.3	1.7	BETTER

new-entry_04.ps
