# Supplementary material for: Structural basis for Z-DNA binding and stabilization by the zebrafish Z-DNA dependent protein kinase PKZ
Source: Nucleic Acids Res. 2013 Aug 23;41(21):9924–33. doi: 10.1093/nar/gkt743 (PMC3834819; doi:10.1093/nar/gkt743)

Residue properties

new-entry

a. Absolute deviation from mean Chi-1 value (excl. Pro)

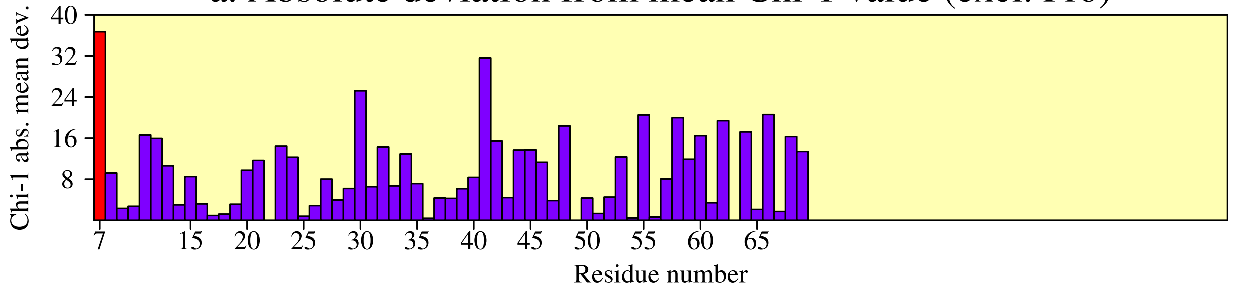

b. Absolute deviation from mean of omega torsion

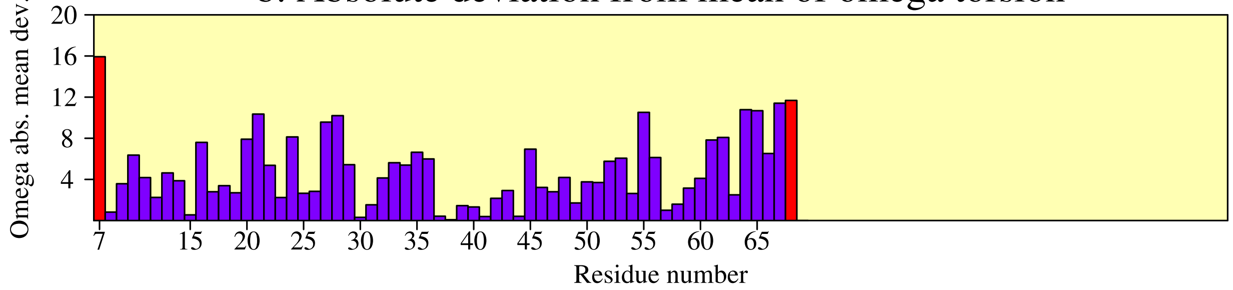

c. C-alpha chirality: abs. deviation of zeta torsion

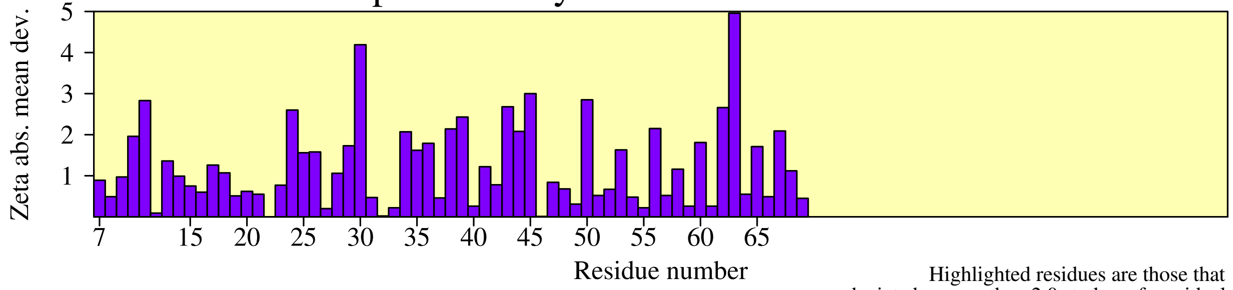

Highlighted residues are those that deviate by more than 2.0 st. devs. from ideal

d. Secondary structure & estimated accessibility

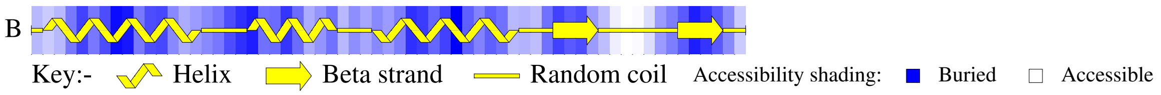

e. Sequence & Ramachandran regions

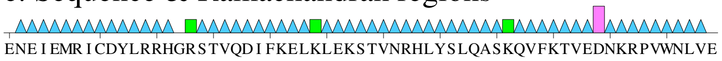

f. Max. deviation (see listing)

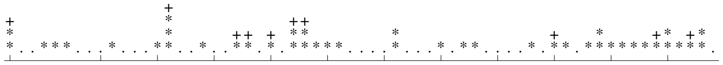

g. G-factors

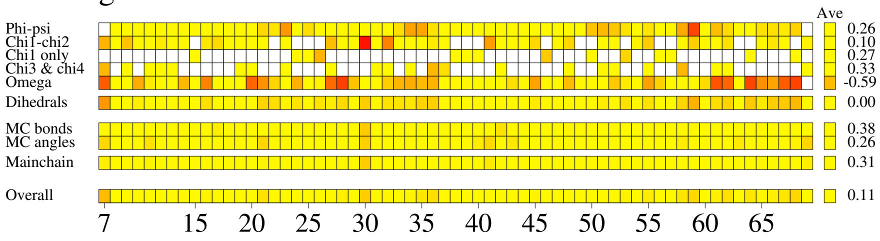

Supplement: Supplementary Data [file supp_gkt743_suppl_data.zip › nar-01793-f-2013-File009.zip!/4LB6/new-entry_06.pdf]
