# Supplementary material for: Structural basis for Z-DNA binding and stabilization by the zebrafish Z-DNA dependent protein kinase PKZ
Source: Nucleic Acids Res. 2013 Aug 23;41(21):9924–33. doi: 10.1093/nar/gkt743 (PMC3834819; doi:10.1093/nar/gkt743)
Supplement: Supplementary Data [file supp_gkt743_suppl_data.zip › nar-01793-f-2013-File009.zip!/4LB6/new-entry_08.pdf]

Main-chain bond angles

new-entry

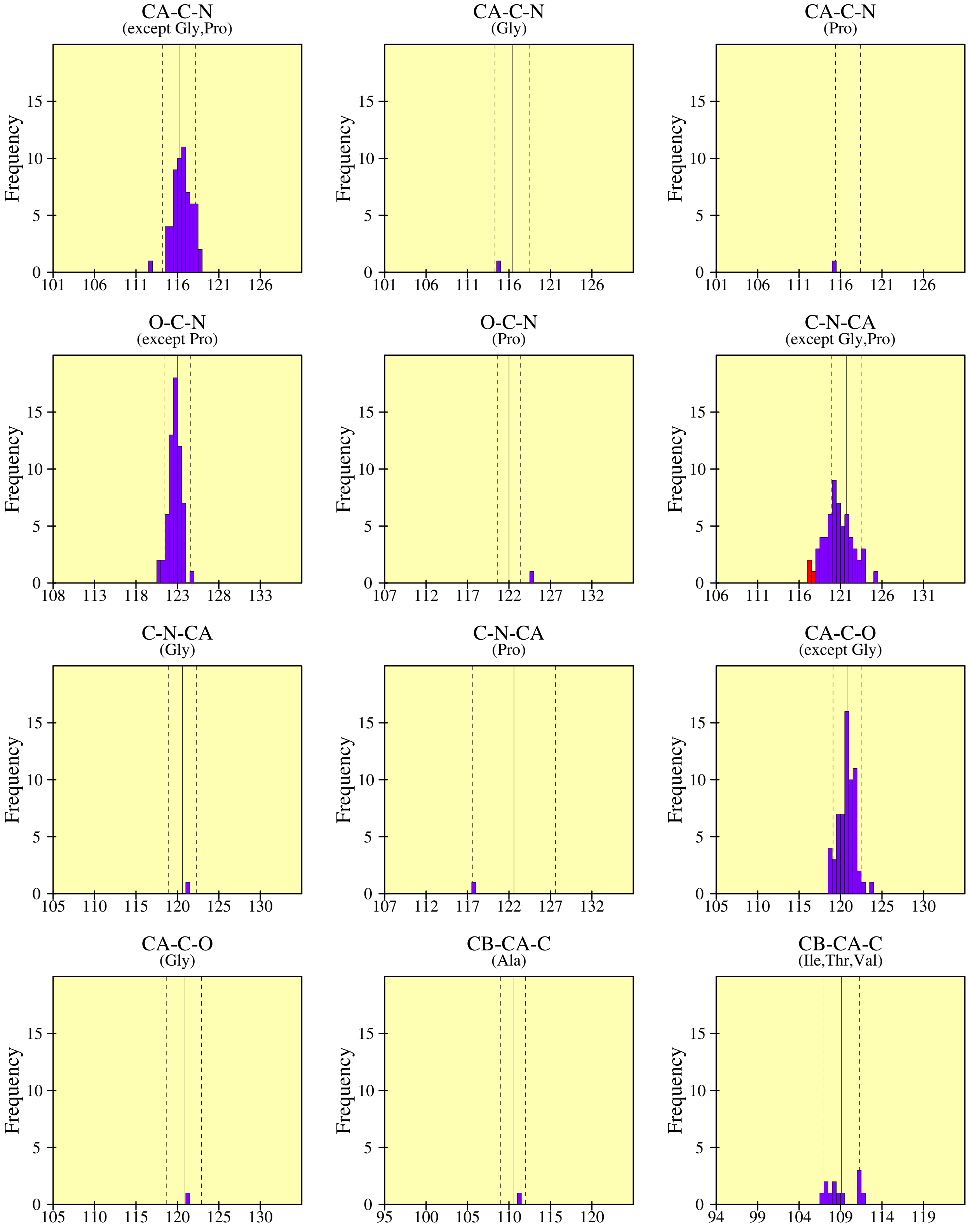

Black bars > 2.0 st. devs. from mean.

Solid and dashed lines represent the mean and standard deviation values as per Engh & Huber small-molecule data.

Main-chain bond angles

new-entry

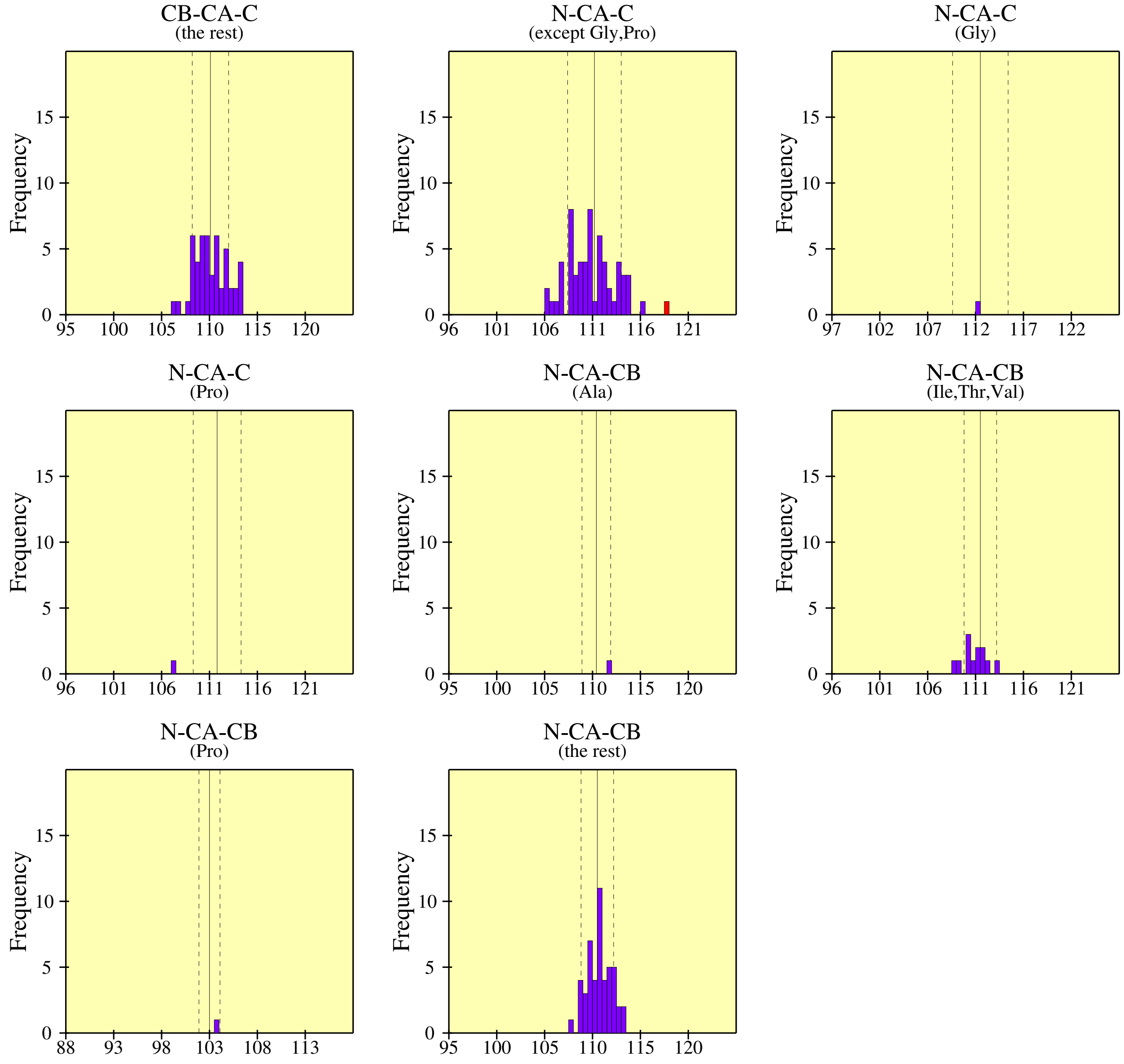

Black bars > 2.0 st. devs. from mean.

Solid and dashed lines represent the mean and standard deviation values as per Engh & Huber small-molecule data.
