# Supplementary material for: Structural basis for Z-DNA binding and stabilization by the zebrafish Z-DNA dependent protein kinase PKZ
Source: Nucleic Acids Res. 2013 Aug 23;41(21):9924–33. doi: 10.1093/nar/gkt743 (PMC3834819; doi:10.1093/nar/gkt743)
Supplement: Supplementary Data [file supp_gkt743_suppl_data.zip › nar-01793-f-2013-File009.zip!/4LB6/new-entry_09.pdf]

RMS distances from planarity

new-entry

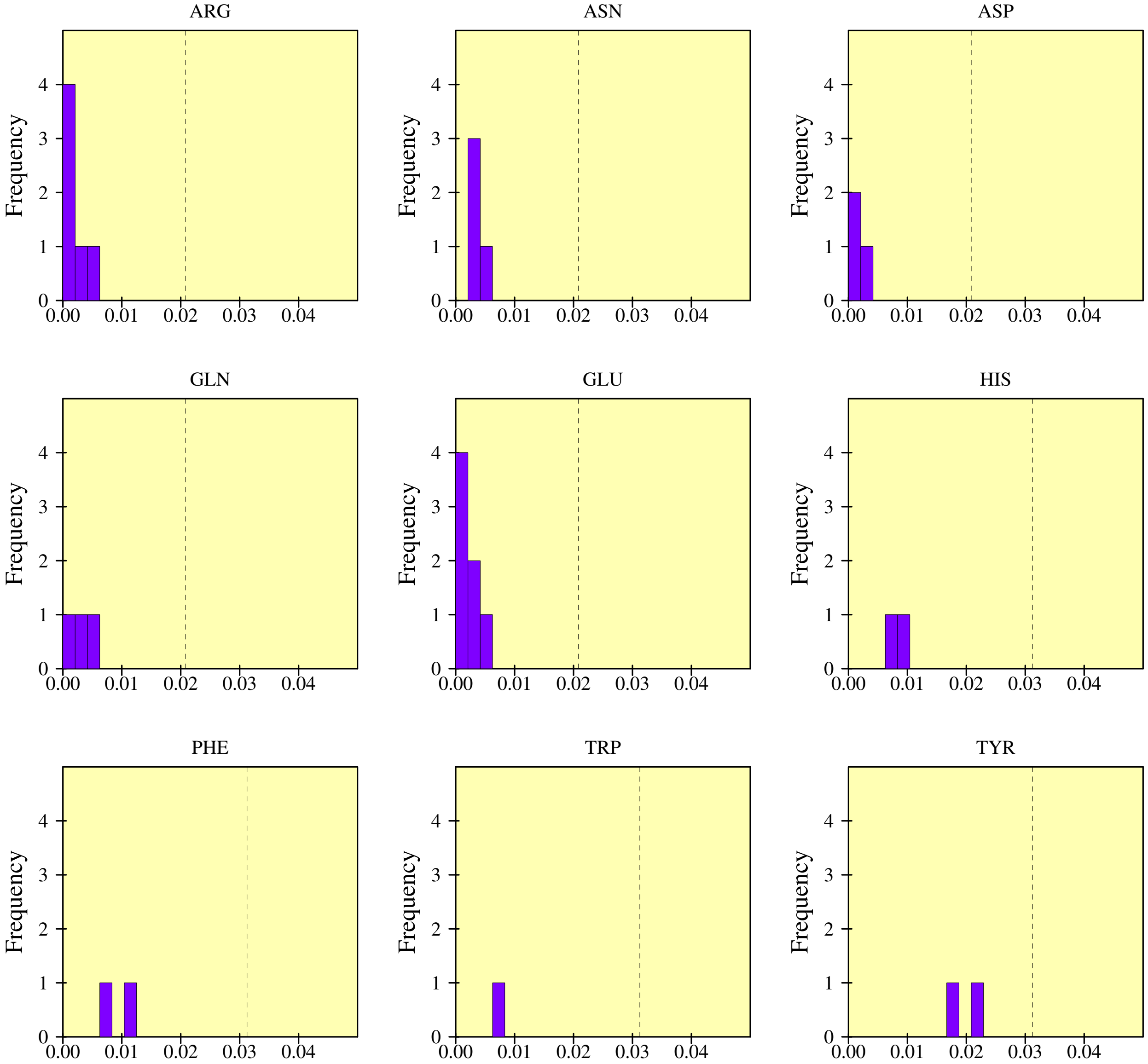

Histograms showing RMS distances of planar atoms from best-fit plane.
Black bars indicate large deviations from planarity: RMS dist > 0.03 for rings, and > 0.02 otherwise.
