# Supplementary material for: Structural basis for Z-DNA binding and stabilization by the zebrafish Z-DNA dependent protein kinase PKZ
Source: Nucleic Acids Res. 2013 Aug 23;41(21):9924–33. doi: 10.1093/nar/gkt743 (PMC3834819; doi:10.1093/nar/gkt743)
Supplement: Supplementary Data [file supp_gkt743_suppl_data.zip › nar-01793-f-2013-File007.zip!/Supplementary Figure Legends.pdf]

Supplementary Figure Legends

Supplementary Figure 1: Possible binding modes of Zalpha domains on Pu/Py repeats. Shown are models of the five possible arrangements of Zalpha domains on a 22 bp duplex DNA, in accordance to Figure 1D. The top row depicts binding sites spaced every 4 bps and the bottom every 6 bps. Zalpha monomers are shown in blue (primary contacts with grey DNA strand) and red (primary contacts with yellow DNA strand) while regions that clash is shown in cyan and pink respectively. Only the (6 – 3/6 + 3) arrangement does not result in steric clashes and can propagate along the DNA helix. In the (6 – 3/6 + 3) model we introduced a 15 aminoacids linker (green) for the pair of monomers that has the most plausible orientation and distance for a C-terminus – N-terminus linkage to represent the Zab binding for DAI, ADAR1 and PKZ. The guanosine contacted by Tyr45 for each monomer is shown in green as surface and indicated the central point of interaction for each monomer.

Supplementary Figure 2: Superposition of DrZalphaPKZ and previously determined Zalpha domain structures. *Dario rerio* PKZ Zα (*DrZ*α_{PKZ}, red, this paper), *Homo sapiens* ADAR1 Zα (*HsZ*α_{ADAR1}, cyan, 1QBJ); *Homo sapiens* ADAR1 Zβ (*HsZ*β_{ADAR1}, black, 1XMK); *Mus musculus* DAI Zα (*MmZ*α_{DAI}, blue, 1J75) *Homo sapiens* DAI Zβ (*HsZ*β_{DAI}, yellow, 3EYI); yaba-like disease virus E3L Zα (*yabaZ*α_{E3L}, green, 1SFU); cyprinid herpesvirus 3 ORF112 Zα dimer (*chv3Z*α_{ORF112}, orange, 4HOB). Zalpha domains are represented as ribbons and an arrow is used to mark the “wing”, the region of the domains which is extended in the case of DrZalpha^{PKZ}. ORF112 forms a domain swapped dimer and both domains are depicted in the figure.

Supplementary Figure 3: Protein – protein interactions among Zalpha domains bound to DNA. Detail of the interactions between monomer B (cyan) and its crystallographic symmetrical molecule (green).
