# Supplementary material for: Structural basis for Z-DNA binding and stabilization by the zebrafish Z-DNA dependent protein kinase PKZ
Source: Nucleic Acids Res. 2013 Aug 23;41(21):9924–33. doi: 10.1093/nar/gkt743 (PMC3834819; doi:10.1093/nar/gkt743)
Supplement: Supplementary Data [file supp_gkt743_suppl_data.zip › nar-01793-f-2013-File007.zip!/Supplementary Figures.pdf]

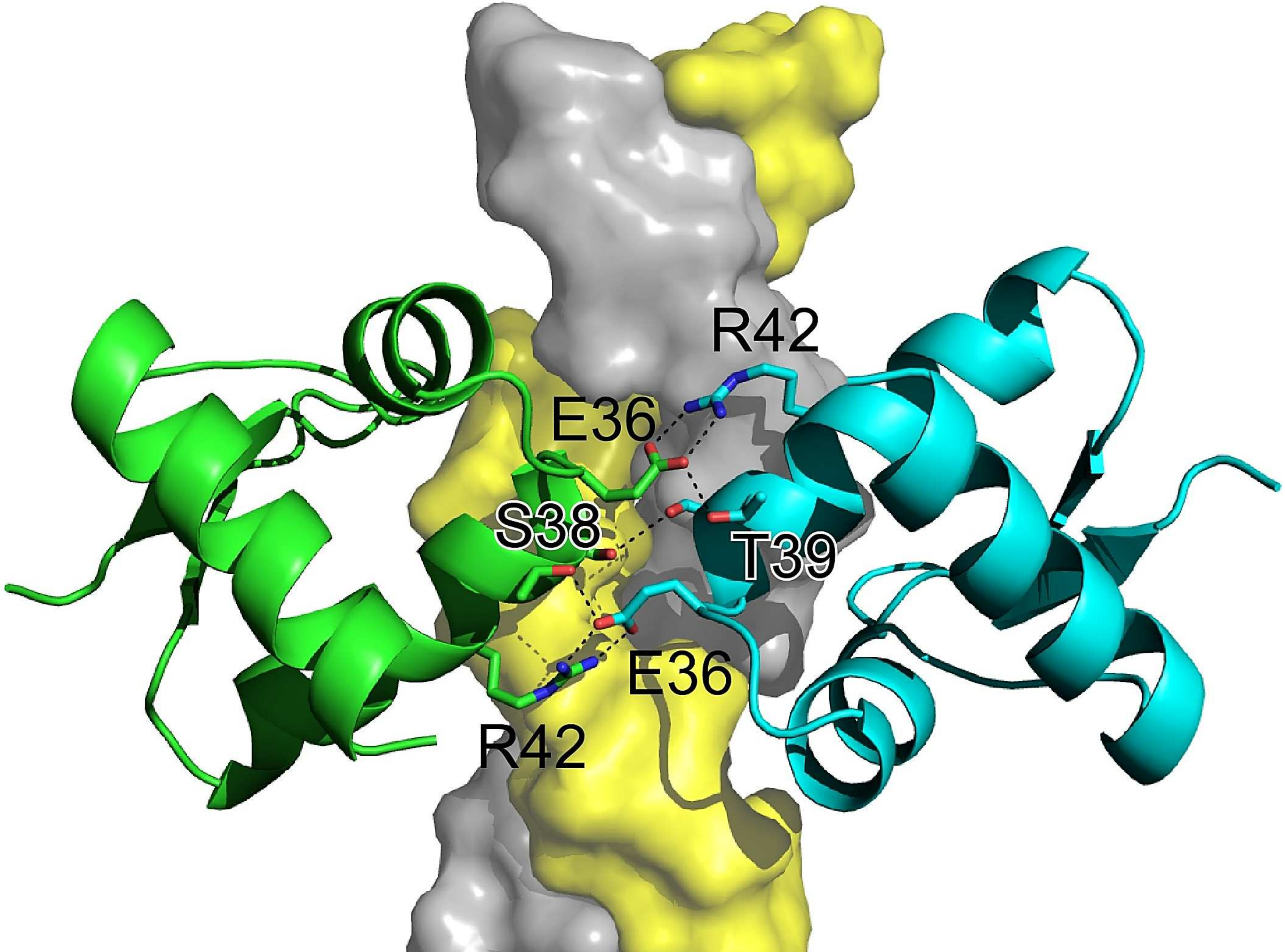
R42
E36
S38
T39
E36
R42

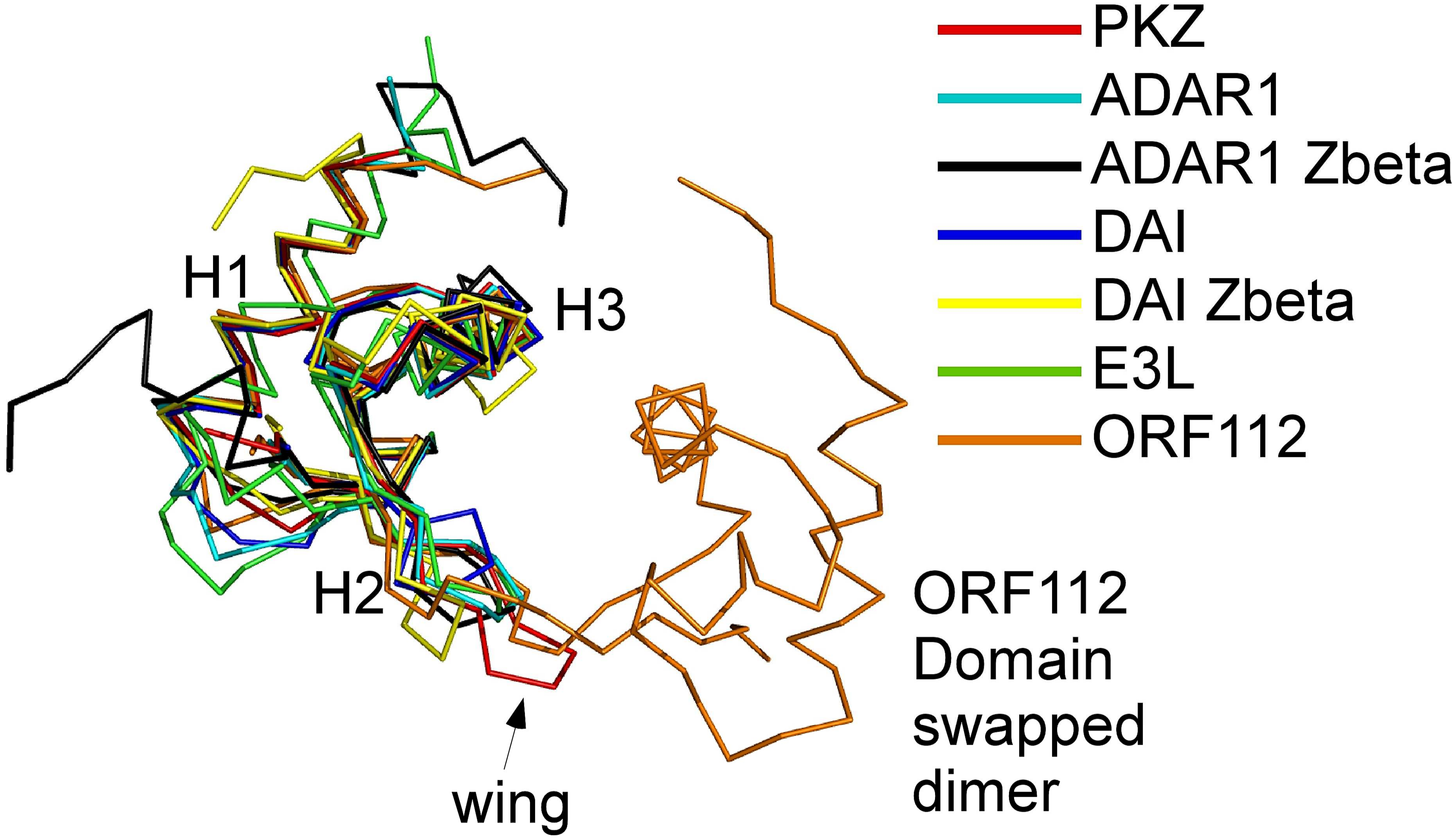
PKZ
ADAR1
ADAR1 Zbeta
DAI
DAI Zbeta
E3L
ORF112
H1
H3
H2
wing
ORF112 Domain swapped dimer

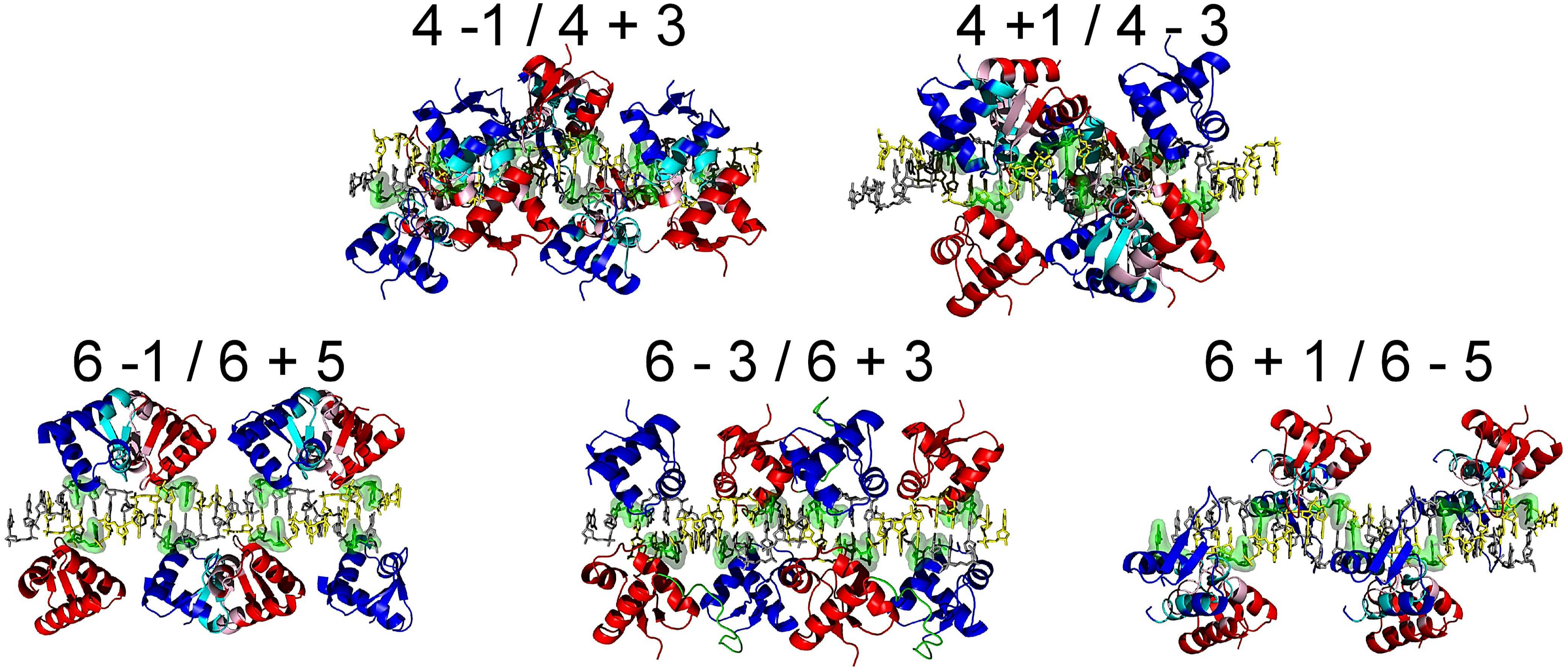
4 -1 / 4 + 3
4 +1 / 4 - 3
6 -1 / 6 + 5
6 - 3 / 6 + 3
6 + 1 / 6 - 5
