# Supplementary material for: Structural basis for Z-DNA binding and stabilization by the zebrafish Z-DNA dependent protein kinase PKZ
Source: Nucleic Acids Res. 2013 Aug 23;41(21):9924–33. doi: 10.1093/nar/gkt743 (PMC3834819; doi:10.1093/nar/gkt743)
Supplement: Supplementary Data [file supp_gkt743_suppl_data.zip › nar-01793-f-2013-File008.zip!/4LB5/new-entry_01.pdf]

PROCHECK

Ramachandran Plot

new-entry

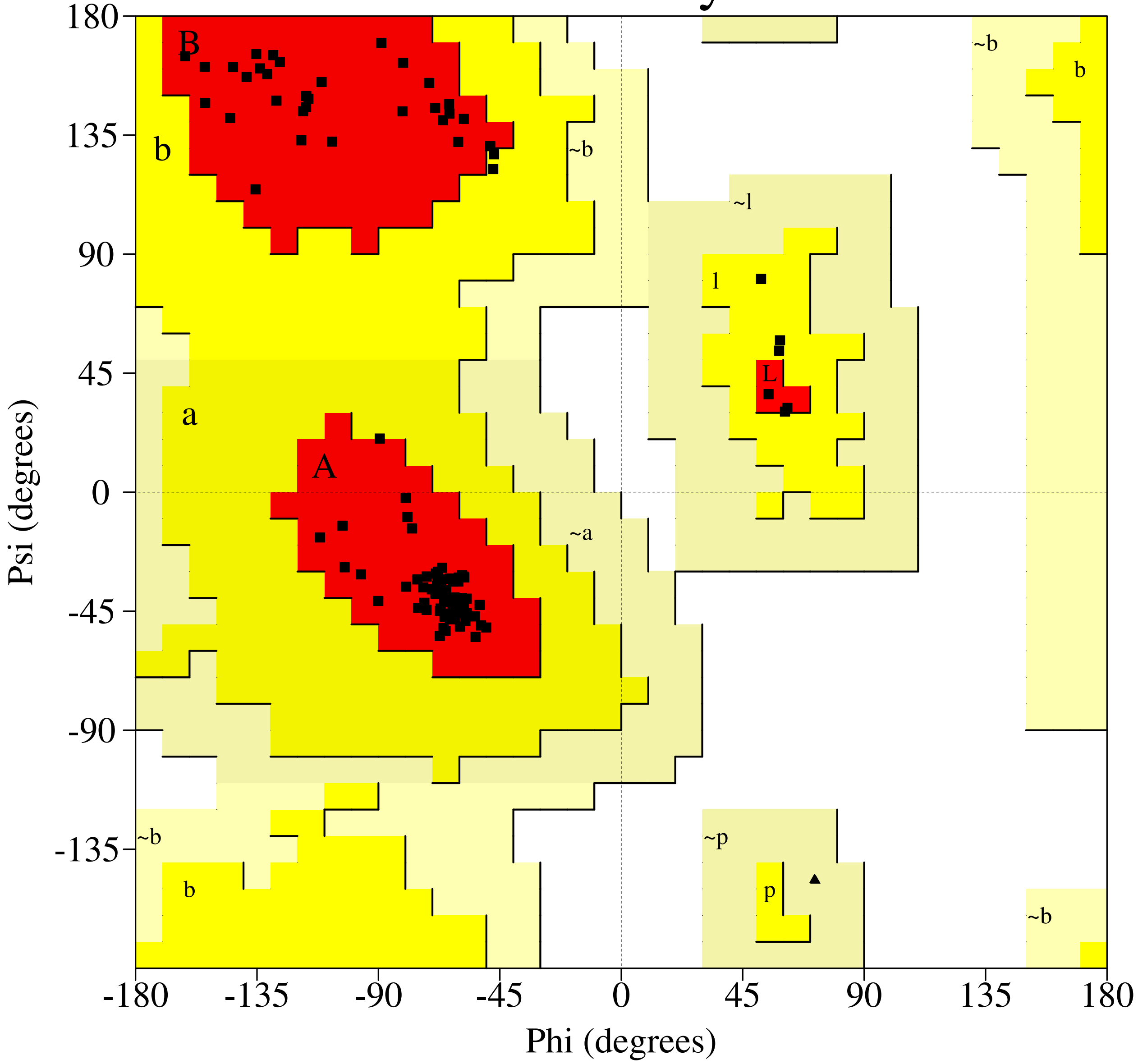

Plot statistics

Residues in most favoured regions [A,B,L]	104	94.5%
Residues in additional allowed regions [a,b,l,p]	6	5.5%
Residues in generously allowed regions [~a,~b,~l,~p]	0	0.0%
Residues in disallowed regions	0	0.0%
	----	------
Number of non-glycine and non-proline residues	110	100.0%
Number of end-residues (excl. Gly and Pro)	6	
Number of glycine residues (shown as triangles)	2	
Number of proline residues	2	

Total number of residues	120	

Based on an analysis of 118 structures of resolution of at least 2.0 Angstroms and R-factor no greater than 20%, a good quality model would be expected to have over 90% in the most favoured regions.
