# Supplementary material for: Structural basis for Z-DNA binding and stabilization by the zebrafish Z-DNA dependent protein kinase PKZ
Source: Nucleic Acids Res. 2013 Aug 23;41(21):9924–33. doi: 10.1093/nar/gkt743 (PMC3834819; doi:10.1093/nar/gkt743)
Supplement: Supplementary Data [file supp_gkt743_suppl_data.zip › nar-01793-f-2013-File008.zip!/4LB5/new-entry_02.pdf]

Ramachandran plots for all residue types

new-entry

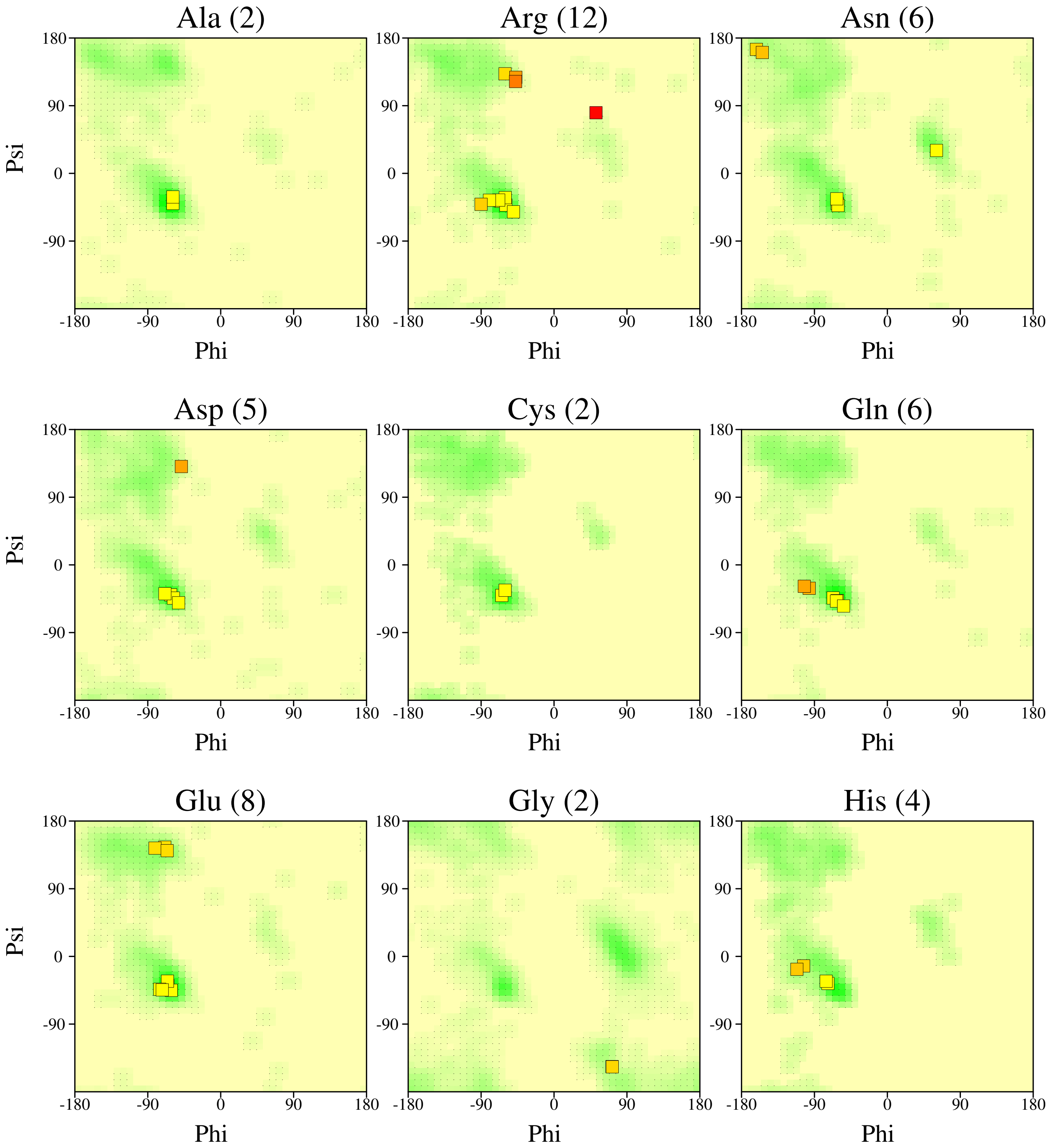

Numbers of residues are shown in brackets. Those in unfavourable conformations (score < -3.00) are labelled.
Shading shows favourable conformations as obtained from an analysis of 163 structures at resolution 2.0A or better.

Ramachandran plots for all residue types

new-entry

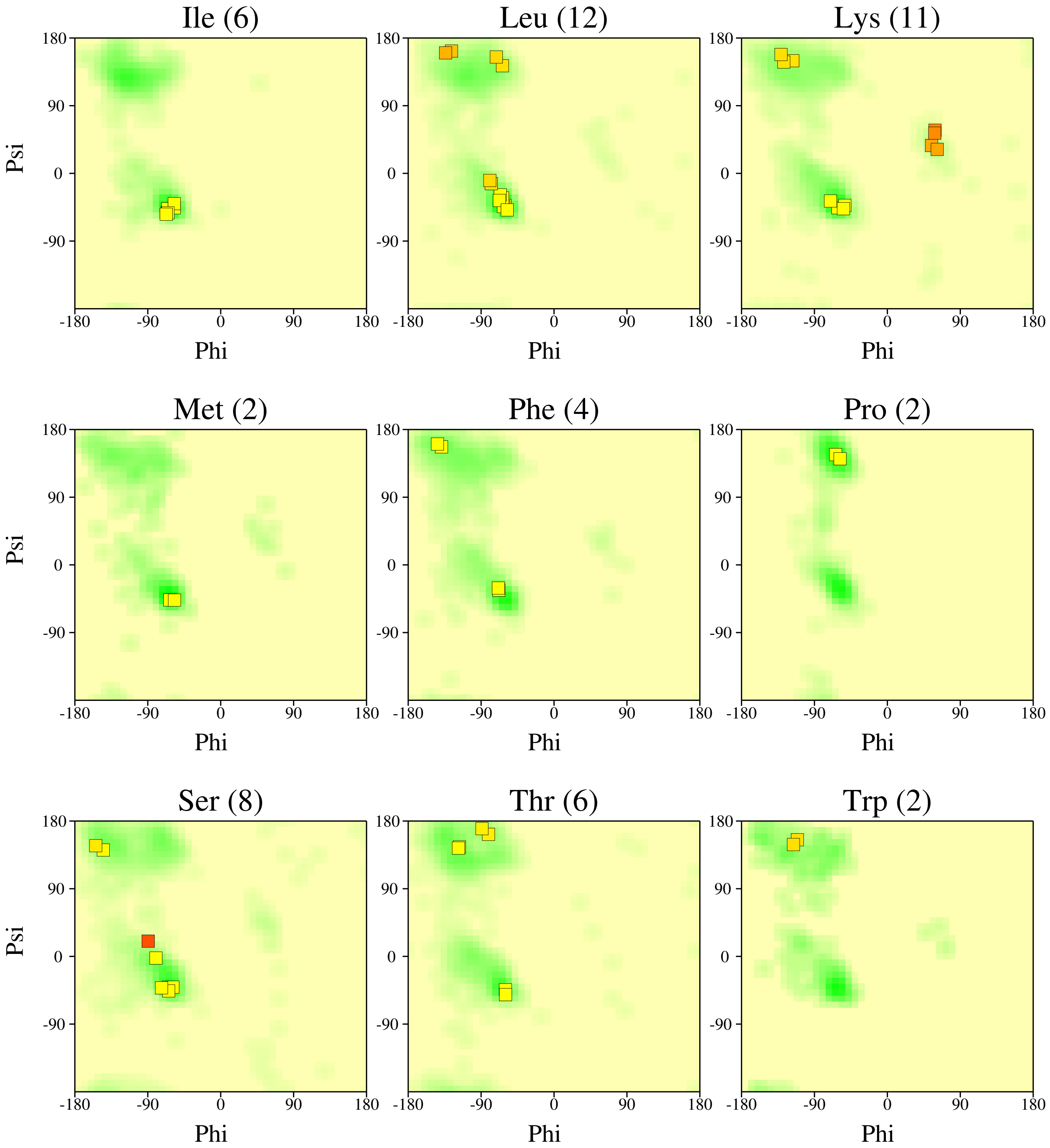

Numbers of residues are shown in brackets. Those in unfavourable conformations (score < -3.00) are labelled.
Shading shows favourable conformations as obtained from an analysis of 163 structures at resolution 2.0A or better.

Ramachandran plots for all residue types

new-entry

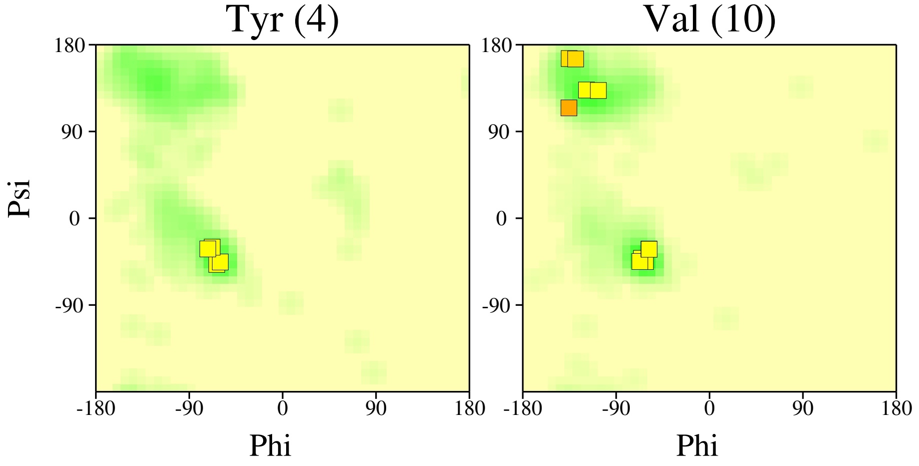

Numbers of residues are shown in brackets. Those in unfavourable conformations (score < -3.00) are labelled.
Shading shows favourable conformations as obtained from an analysis of 163 structures at resolution 2.0A or better.
