# Supplementary material for: Structural basis for Z-DNA binding and stabilization by the zebrafish Z-DNA dependent protein kinase PKZ
Source: Nucleic Acids Res. 2013 Aug 23;41(21):9924–33. doi: 10.1093/nar/gkt743 (PMC3834819; doi:10.1093/nar/gkt743)
Supplement: Supplementary Data [file supp_gkt743_suppl_data.zip › nar-01793-f-2013-File008.zip!/4LB5/new-entry_04.pdf]

PROCHECK

Main-chain parameters

new-entry

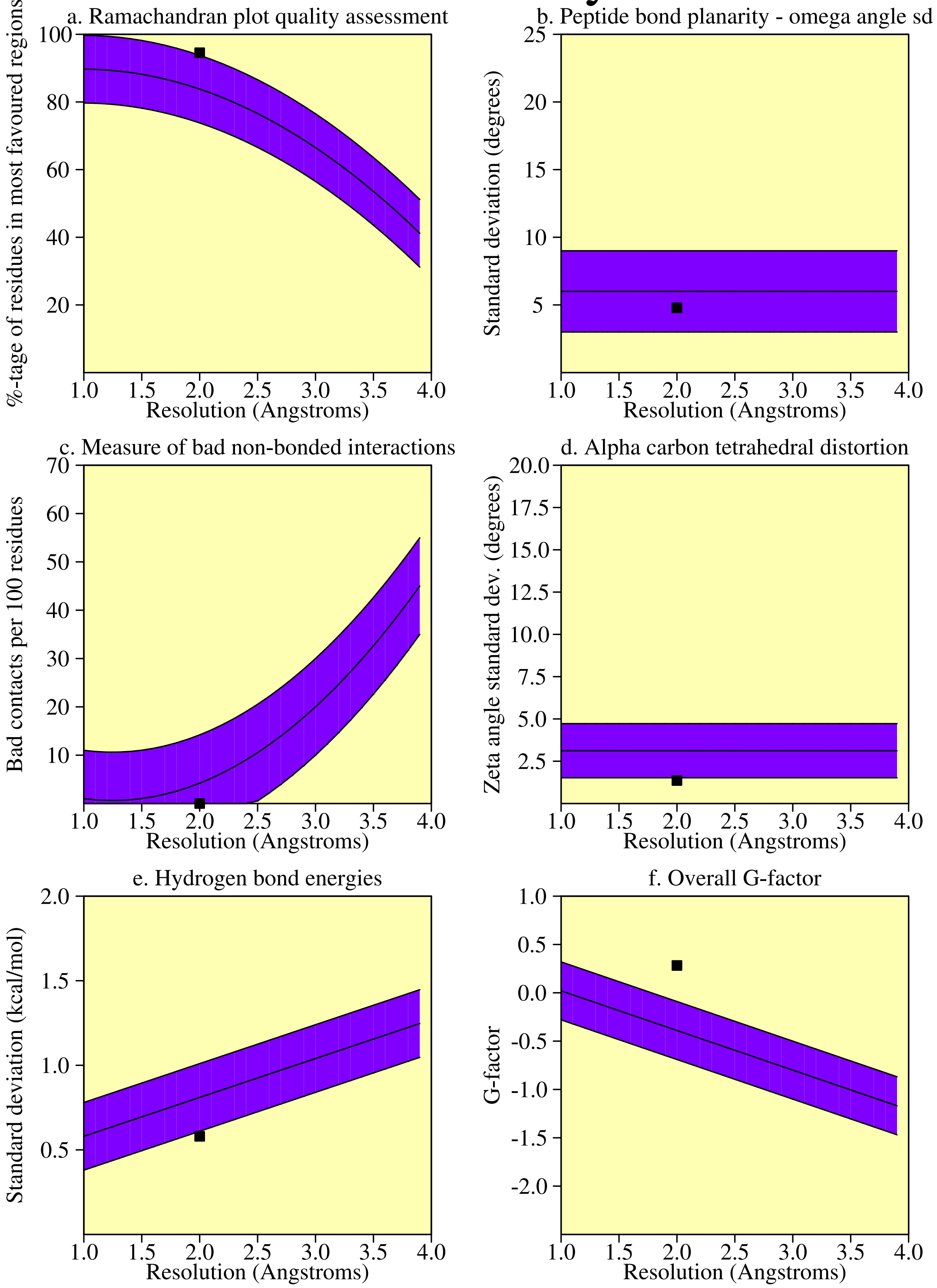

Plot statistics

Stereochemical parameter	No. of data pts	Parameter value	Comparison values Typical value	Band width	No. of band widths from mean	
a. %-tage residues in A, B, L	110	94.5	83.8	10.0	1.1	BETTER
b. Omega angle st dev	117	4.8	6.0	3.0	-0.4	Inside
c. Bad contacts / 100 residues	0	0.0	4.2	10.0	-0.4	Inside
d. Zeta angle st dev	118	1.4	3.1	1.6	-1.1	BETTER
e. H-bond energy st dev	79	0.6	0.8	0.2	-1.1	BETTER
f. Overall G-factor	120	0.3	-0.4	0.3	2.2	BETTER
