# Supplementary material for: Structural basis for Z-DNA binding and stabilization by the zebrafish Z-DNA dependent protein kinase PKZ
Source: Nucleic Acids Res. 2013 Aug 23;41(21):9924–33. doi: 10.1093/nar/gkt743 (PMC3834819; doi:10.1093/nar/gkt743)
Supplement: Supplementary Data [file supp_gkt743_suppl_data.zip › nar-01793-f-2013-File008.zip!/4LB5/new-entry_05.pdf]

PROCHECK

Side-chain parameters

new-entry

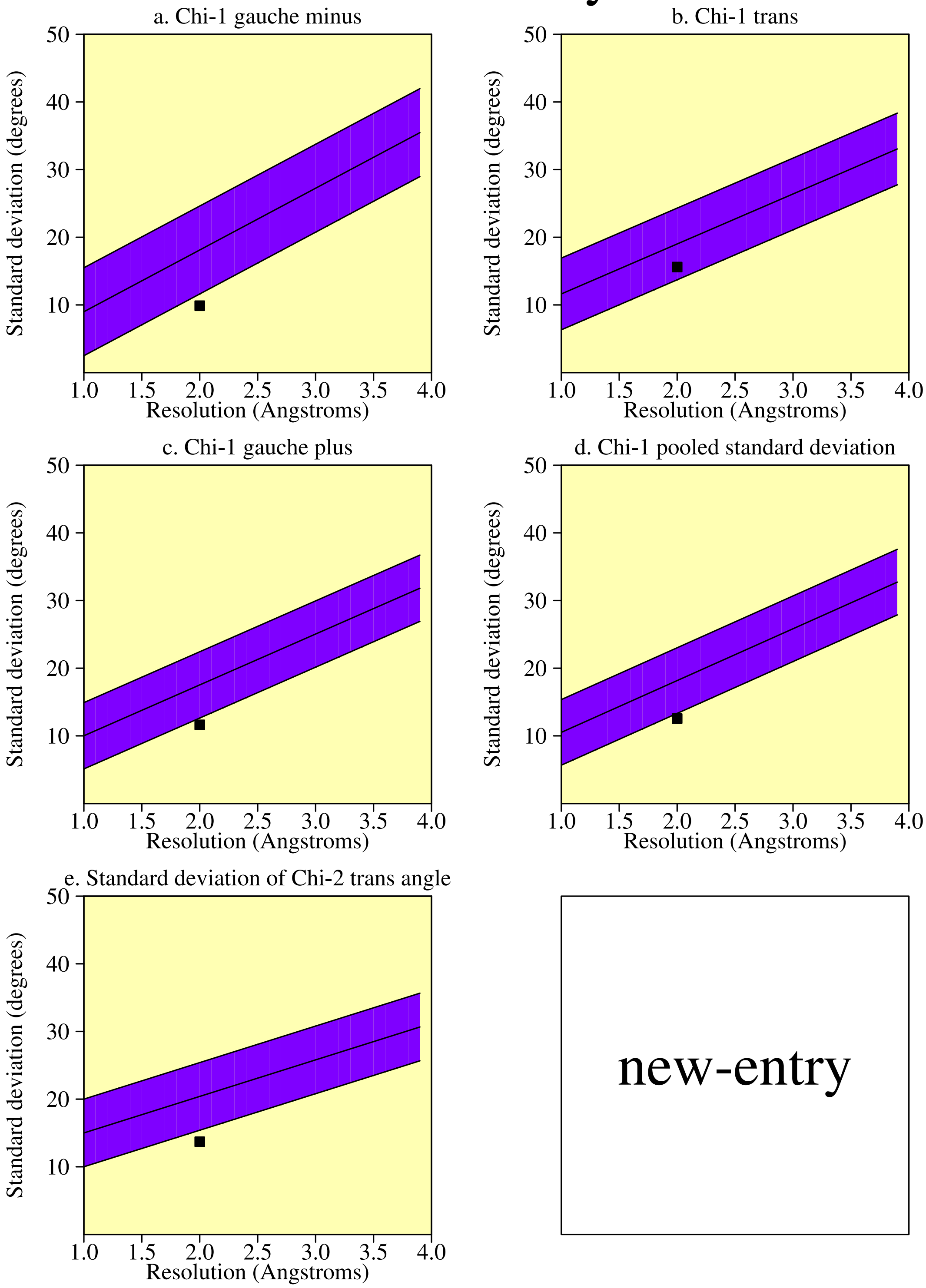

Plot statistics

Stereochemical parameter	No. of data pts	Parameter value	Comparison values Typical value	Band width	No. of band widths from mean	
a. Chi-1 gauche minus st dev	16	9.9	18.1	6.5	-1.3	BETTER
b. Chi-1 trans st dev	29	15.6	19.0	5.3	-0.6	Inside
c. Chi-1 gauche plus st dev	67	11.6	17.5	4.9	-1.2	BETTER
d. Chi-1 pooled st dev	112	12.6	18.2	4.8	-1.2	BETTER
e. Chi-2 trans st dev	44	13.7	20.4	5.0	-1.3	BETTER
