# Supplementary material for: Structural basis for Z-DNA binding and stabilization by the zebrafish Z-DNA dependent protein kinase PKZ
Source: Nucleic Acids Res. 2013 Aug 23;41(21):9924–33. doi: 10.1093/nar/gkt743 (PMC3834819; doi:10.1093/nar/gkt743)
Supplement: Supplementary Data [file supp_gkt743_suppl_data.zip › nar-01793-f-2013-File008.zip!/4LB5/validation_report.rtf]

Validation Report


Thank you for using PDB.  The following geometrical and stereochemical 
features have been calculated for your structure:

    CLOSE CONTACTS
    --------------

==> Close contacts in same asymmetric unit.  Distances smaller than 2.2 
    Angstroms are considered as close contacts.                

      none


==> Close contacts based on crystal symmetry.  Distances smaller than 2.2
    Angstroms are considered as close contacts.

      none


    BOND DISTANCES AND ANGLES
    -------------------------

==> Bond and angle checks are performed by first computing the average rms
    error for all bonds and angles relative to standard values for nucleotide
    units [L. Clowney et al., Geometric Parameters in Nucleic Acids: Nitrogenous
    Bases, J.Am.Chem.Soc. 1996, 118, 509-518; A. Gelbin et al., Geometric
    Parameters in Nucleic Acids: Sugar and Phosphate Constituents, J.Am.Chem.Soc.
    1996, 118, 519-529] and amino acid units [R.A. Engh and R. Huber, Structure
    quality and target parameters, International Tables for Crystallography,
    Volume F, 2001, 382-392]. Any bond or angle which deviates from the
    dictionary values by more than six times this computed rms error is 
    identified as an outlier.


    *** Covalent Bond Lengths:

    The overall RMS deviation for covalent bonds relative to the standard 
    dictionary is   0.007 Angstroms

    All covalent bonds lie within a 6.0*RMSD range about the 
    standard dictionary values.

    *** Covalent Angle Values:

    The overall RMS deviation for covalent angles relative to the standard 
    dictionary is    1.1 degrees.

    The following table contains a list of the covalent bond angles
    greater than 6 times standard deviation.


 Deviation  Residue  Chain  Sequence  AT1  -  AT2  -  AT3    Bond    Dictionary  Standard
             Name     ID     Number                          Angle      Value    Deviation
-------------------------------------------------------------------------------------------
     1.8     DC       C        1      N1   -  C1'  -  O4'    110.1     108.3        0.3
     2.3     DC       C        7      N1   -  C1'  -  O4'    110.6     108.3        0.3
    -5.3     DG       C       10      N9   -  C1'  -  O4'    102.7     108.0        0.7


    3 out of total 1787 bond angles (0.168%) have greater than 6 times
    standard deviation.


    TORSION ANGLES
    --------------

    The torsion angle distributions have been checked.  Refer to the
    Procheck and/or Nucheck results on the PDB Validation Report page.


==> The following table contains a list of torsion angles outside the expected
    Ramachandran regions [GJ. Kleywegt and TA. Jones, PHI/PSI-chology:
    Ramachandran Revisited, Structure  1996, 4, 1395 - 1400].

      Residue  Chain  Sequence              PSI       PHI
        ARG      A       62               80.64     51.79

    CHIRALITY
    ---------

    The chirality has been checked. O1P, O2P, and hydrogen atoms which do not
    follow the conventions defined by IUBMB (Liebecq, C. Compendium of 
    Biochemical Nomenclature and Related Documents, 2nd ed.; Portland Press:
    London and Chapel Hill, 1992) and IUPAC (J.L. Markley, A. Bax,
    Y. Arata, C.W. Hilbers, R. Kaptein, B.D. Sykes, P.E. Wright and K. Wuthrich,
    Recommendations for the Presentation of NMR Structures of Proteins and
    Nucleic Acids, Pure & Appl. Chem., Vol. 70, pp. 117-142, 1998) will be  
    standardized at the time of processing; there is no need to change these 
    labels in your coordinate file.  Any other stereochemical violations are
    listed below.

Chain  Residue  Residue  Chiral       Chiral Neighbors      Original
        Name    Number   Center                             Atom Name
-----  -------  ------- --------  ------------------------  ---------
C        DG       12    P         OP2                       
C        DG       12    P         OP1                       

    SOLVENT 
    -------

    The following solvent molecules are further than 3.5 Angstroms away from
    macromolecule atoms in the asymmetric unit that are available for 
    hydrogen bonding. Solvent molecules in extended hydration shells 
    separated by 3.5 Angstroms or less are not listed.
    
HETATM 1318  O   HOH D  78     -26.530  15.693 -22.436  1.00 47.56      DIST =   3.92 A
HETATM 1353  O   HOH D 124     -36.708  13.209   6.929  1.00 62.74      DIST =   3.78 A

    We have replaced the coordinates for solvent molecules which could be
    translated back into the asymmetric unit. Please review all solvent
    molecules in your file and contact us if you have any serious objections.


    

SEQUENCE WARNING: Residue A VAL 57 and Residue A LYS 61 are not properly linked:
 C-N bond distance is 6.75 A.

    MISSING ATOMS
    -------------

==> The following residues have missing atoms:                        

     RES MOD#C SEQ          ATOMS

     LYS(    A  31)          CD   CE   NZ 
     LYS(    A  34)          CG   CD   CE   NZ 
     LYS(    A  51)          CE   NZ 
     LYS(    A  61)          CE   NZ 
     LYS(    B  34)          CE   NZ 
     GLU(    B  58)          CG   CD  OE1  OE2 
     GLU(    B  69)          CD  OE1  OE2 
      DT(    C   0)         O5'  C5'  C4'  O4'  C3'  C2'  C1'   N1   C2   O2 
                             N3   C4   O4   C5   C7   C6
